# Recent progress of photocatalysts based on tungsten and related metals for nitrogen reduction to ammonia

**DOI:** 10.3389/fchem.2022.978078

**Published:** 2022-08-22

**Authors:** Xiangchao Hui, Lijun Wang, Zhibo Yao, Leiduan Hao, Zhenyu Sun

**Affiliations:** State Key Laboratory of Organic-Inorganic Composites, College of Chemical Engineering, Beijing University of Chemical Technology, Beijing, China

**Keywords:** photocatalysis, nitrogen reduction, ammonia synthesis, tungsten, semiconductor

## Abstract

Photocatalytic nitrogen reduction reaction (NRR) to ammonia holds a great promise for substituting the traditional energy-intensive Haber–Bosch process, which entails sunlight as an inexhaustible resource and water as a hydrogen source under mild conditions. Remarkable progress has been achieved regarding the activation and solar conversion of N_2_ to NH_3_ with the rapid development of emerging photocatalysts, but it still suffers from low efficiency. A comprehensive review on photocatalysts covering tungsten and related metals as well as their broad ranges of alloys and compounds is lacking. This article aims to summarize recent advances in this regard, focusing on the strategies to enhance the photocatalytic performance of tungsten and related metal semiconductors for the NRR. The fundamentals of solar-to-NH_3_ photocatalysis, reaction pathways, and NH_3_ quantification methods are presented, and the concomitant challenges are also revealed. Finally, we cast insights into the future development of sustainable NH_3_ production, and highlight some potential directions for further research in this vibrant field.

## 1 Introduction

As the cornerstone of modern civilization, ammonia (NH_3_) is an incontrovertible raw material for modern industry and agriculture, which plays a crucial role in human survival and economic growth. Moreover, due to its high hydrogen content (17.7 wt%), gravimetric energy density (3 kWh kg^−1^) and easy liquification (−33°C under atmospheric pressure), NH_3_ also serves as a useful commodity for chemicals used in industries and as a carbon-free clean energy carrier (Guo and Chen, 2017). NH_3_ is by far predominantly fabricated via the energy- and capital-intensive Haber-Bosch process requiring extreme reaction conditions of 300–500°C and 15–25 MPa, which gives rise to excessive consumption of feedstocks and consequently high CO_2_ emissions. Therefore, exploring and developing renewable, environment-friendly, and green routes to yield NH_3_ is desirable. Photocatalytic N_2_ fixation is perceived as an alternative sustainable strategy for facile, cost-effective ammonia production using water and nitrogen gas under ambient conditions. There is enormous, acknowledged and untapped potential in this emerging field. Considering that nitrogen reduction reaction (NRR) coupled with water oxidation is a thermodynamically uphill reaction, solar energy is hence crucial to induce incoming photons generating electronic charge carriers to initiate the catalytic reaction. Since the pioneering research by Schrauzer and co-workers embarked on the photocatalytic reduction of N_2_ to NH_3_ by employing TiO_2_-based photocatalysts (Schrauzer and Guth, 1977), explorative efforts in this sector have been exerted to develop novel photocatalysts with high efficiency, especially in recent years (Schrauzer and Guth, 2002). The initial attempts are currently expanded to widen the scope of investigated materials and their modifications, as well as effective strategies of tuning crystalline phase, surface defects, heteroatom doping, surface modification and/or heterostructure construction for enhancing photocatalytic performances. The photochemical process underpins the terms of selectivity, efficiency, and low operational cost for the production of NH_3_ toward the practical implementation at relatively ambient conditions using solar energy. Despite the huge potentials, the photocatalytic NH_3_ production still falls far short of the ideal of being commercialized, which results from weak adsorption/activation of the nonpolar N≡N triple bond of N_2_ (941 kJ mol^−1^), inefficient light absorption, and poor photo-induced charge separation (Shen et al., 2021).

Design and development of efficient photocatalysts hold the key to achieve improved performance of photocatalytic NRR. To date, several prior articles (Chen et al., 2018; Shi et al., 2019; Shuai Zhang et al., 2019; Huang et al., 2020; Chen-Xuan Xu et al., 2021; Lee et al., 2021; Shen et al., 2021; Tong Xu et al., 2021) have outlined the recent advancements of photoreduction of N_2_ to NH_3_ and photocatalysts engineering strategies. Transition metals, especially the early transition metals (e.g., tungsten, molybdenum, vanadium etc.), which possess both empty orbitals and abundant *d*-orbital electrons as well as suitable bandgap energies, could activate dinitrogen molecules through *σ*-donation/*π*-backdonation effects, showing huge potential for applications as photocatalysts for photocatalytic NRR. However, few reviews, to the best of our knowledge, have hitherto summarized tungsten and related metals photocatalysts together with a specific focus on strategies to enhance their performances. This review elaborated the state-of-the-art understanding of the basic principles of NRR photocatalysis, reaction mechanisms, thermodynamic limits, and enforceable protocols involved in the overall photochemical processes. We comprehensively discuss recent progress over semiconductors containing tungsten (W), molybdenum (Mo), cobalt (Co), vanadium(V), tantalum (Ta), niobium (Nb), rhenium (Re), zirconium (Zr), hafnium (Hf), and their major advantages as to photocatalysis activity. Furthermore, we emphasize several strategies to improve the photocatalytic performance and also highlight the challenges and future directions for sustainable NH_3_ production.

## 2 Fundamentals for photochemical NRR

### 2.1 Properties of N_2_ molecules

**FIGURE 1:**
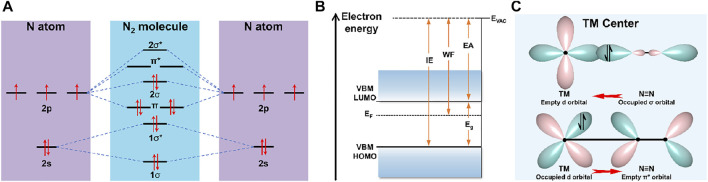
**(A)** Diagram of N atomic orbitals and N_2_ molecular orbitals. **(B)** Energy diagram for N_2_. *E*_VAC_, *E*_g_, WF, IE, EA, and E_F_ represent vacuum level, energy gap, work function, ionization energy, electron affinity, and Fermi level, respectively. **(C)** Schematic illustration of N_2_ bonded to TMs.

Molecular dinitrogen possesses a triple bond between the nitrogen atoms and a non-bonding pair of electrons on each atom. Atomic nitrogen has 5 valence electrons and 4 valence orbitals (2*s*, 2*p*_*x*_, 2*p*_*y*_, and 2*p*_*z*_), whereas hybridization of the *s-p* atomic orbitals of N_2_ consists of four bonding orbitals (two *σ* and two *π* orbitals) and four antibonding orbitals (two σ* and two π* orbitals). The electrons from the *π* and 2*σ* orbitals are shared to form N≡N bond leaving these from 1σ* and 1*σ* orbitals the non-bonding electron pairs (Figure 1A) (Kitano et al., 2012). Hence, the large energy gap of 10.82 eV between the highest occupied molecular orbital (HOMO) and lowest unoccupied molecular orbital (LUMO) seriously hinders electron injection into N_2_ antibonding orbitals (Jia and Quadrelli, 2014). A strong N≡N bond energy (945 kJ mol^−1^) and first-bond breaking energy (410 kJ mol^−1^) render N_2_ molecules extremely thermodynamically stable (Gambarotta and Scott, 2004), meanwhile, N_2_ molecules are chemically inert by virtue of high ionization energy of 15.85 eV and negative electron affinity of −1.9 eV (Figure 1B). Therefore, adsorption and dissociation of N_2_ with weak polarizability and lacking dipole moment are widely regarded to be the rate-determining steps of NRR (Guo et al., 2018; Liang Yang et al., 2020; Yi-Fei Zhang et al., 2020). Both computational and experimental works have demonstrated that transition metal-based materials interact strongly with N_2_ through the formation of N–metal bonds, thanks to the empty *d* orbitals in the transition metals (TMs) accepting the lone pair electrons of N_2_ and back donating *d-p* electrons into the anti-binding orbitals of N_2_ based on an “acceptance-donation” protocol, whereby the triple bond can be weakened and activated to facilitate the bond dissociation (Figure 1C). Some advances have demonstrated the incomparable advantages of *d* block compounds, but this realm remains elusive.

### 2.2 Principles and mechanisms of photochemical NRR to NH_3_

**FIGURE 2:**
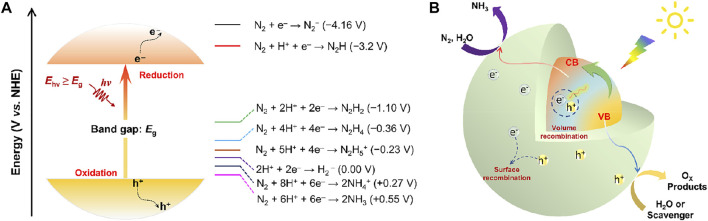
**(A)** Schematic energy diagram related to the reduction of N_2_ to NH_3_. **(B)** Schematic illustration of the complete photocatalytic NH_3_ synthesis over semiconductor-based photocatalysts.

Ideally, photocatalytic NRR into NH_3_ involves transfer of 6 protons and 6electrons, which is a strong endothermic reaction with stoichiometric O_2_ production formed by water oxidation (overall reaction: N_2_ (g) + 3H_2_O (l) ↔ 2NH_3_ (g) + 3/2O_2_ (g), ∆*G* = +7.03 eV) (Medford and Hatzell, 2017). The photochemical NRR requires a potential of at least 1.17 eV per electron (Comer and Medford, 2018), which is initiated by the absorption of photons with an energy higher than the energy bandgap (*E*_g_) of a semiconductor photocatalyst to generate photoexcited electron-hole pairs and an energy >1.17 eV demanded for the overall NH_3_ production (Figure 2A). Upon photo-irradiation with sufficient energy supply, electrons (e^−^) leap into the conduction band (CB) with simultaneous generation of holes (h^+^) at valence band (VB), thus triggering the reduction of N_2_ to NH_3_ with photo-excited electrons and water oxidation with created holes (Linsebigler et al., 1995). The photoinduced separation of charge carriers is the prerequisite for all semiconductor photocatalysis. However, the migration and separation of photoexcited electron-hole pairs to the semiconductor surface active sites to participate in the redox reactions competes with their recombination in the bulk and on the surface. Eventually, the reaction products desorb from the photocatalyst surface and are transferred to the medium to close the cycle (Figure 2B). Note that the NRR is an uphill reaction. As a consequence, the CB and VB positions of a photocatalyst must bestride the reduction potential of N_2_ and the oxidation potential of H_2_O. Theoretically, the bottom of the CB should be more negative than the reduction potential of N_2_/N_2_H (−3.2 V vs. normal hydrogen electrode, NHE), while the top of VB must be beyond the oxidation potential of H_2_O/O_2_ (+1.23 V vs. NHE). Satisfying both of the above requirements with a singular conventional semiconductor seems to be incompatible, which is unfavorable for harvesting most of the light across the solar spectrum. Since a considerable number of electron-hole pairs recombine inside or on the surface of the catalyst with a rather fast kinetics rate (Zhang et al., 2012), or dissipate in the form of heat or light energy, thus resulting in a decrease of reaction efficiency. Accordingly, most of the reported catalysts for photocatalytic NH_3_ production still suffer from low light utilization, fast recombination of photoexcited electron-hole pairs, poor N_2_ adsorption/activation, and sluggish electron-to-N_2_ transfer kinetics, hobbling the overall solar-to-ammonia conversion efficiency. Additionally, the insufficient stability of the photocatalysts is another serious issue. Many semiconductors could undergo photo-corrosion upon light irradiation, which can be induced by the photo-generated electrons or holes, thus leading to degradation of NRR performance and inhibiting the long-time photocatalytic ammonia synthesis.

**FIGURE 3:**
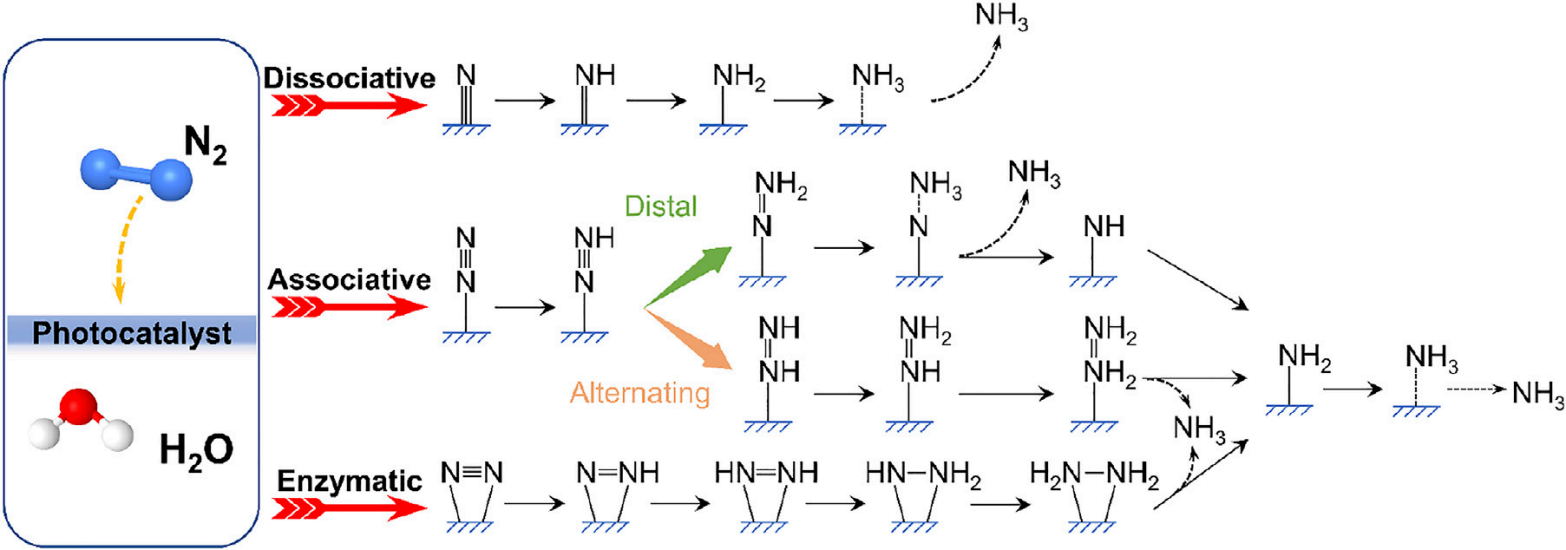
Schematic representation of possible pathways for N_2_ fixation to NH_3_ on catalyst surfaces.

The current well-established mechanisms for photocatalytic NRR can be roughly divided into the following: dissociative, associative or enzymatic pathways as shown in Figure 3 (van der Ham et al., 2014; Hao Li et al., 2016; Shipman and Symes, 2017). For the dissociative pathway, the breaking of the N≡N bond precedes the hydrogenation process, followed by the stepwise protonation of the adsorbed nitrogen atoms to form NH_3_, analogous to the reaction mechanism of the industrial Haber-Bosch process (Garden and Skulason, 2015), which is however unfavorable for N_2_ photoreduction to NH_3_ resulting from the large external energy input required for the cleavage of the N≡N triple bond (Shipman and Symes, 2017). According to the different hydrogenation sequences, the associative N_2_ reduction mechanism follows either distal pathway or alternating pathway. In the distal associative pathway, the nitrogen atom far from the adsorption site is protonated successively before generating the first NH_3_ molecule, leaving another N atom to yield the second NH_3_ molecule. Conversely, in the alternate pathway, the hydrogenation reaction occurs alternately at two nitrogen atoms, each of which could react with injected electrons and protons, forming key intermediates such as metal-bound diazene (HN = NH) and reaction byproducts such as N_2_H_4_ (Bo et al., 2021). As for the enzymatic pathway, the 2 N atoms of the nitrogen molecule are simultaneously adsorbed by the active center of the catalyst, anchored on the catalyst surface via the “side-on” configuration, and then hydrogenated (Hinnemann and Norskov, 2006; Xiao-Fei Li et al., 2016). The proposal that gained the widest support was that photocatalytic NH_3_ synthesis follows associative pathways, during which the adsorption and activation of N_2_ and then the transfer of photogenerated electrons from the photocatalyst to N_2_ provide a lower reaction energy barrier for the dissociation of N≡N triple bonds (Montoya et al., 2015). The activation of N_2_ involves the formation of a coordinate bond with the active site proceeded on a catalytic surface, and the subsequent electron transfer and protonation are the keys to weakening the N≡N bonding energy (Jacobsen et al., 2001). Different mechanisms have been proposed, but deep mechanistic understanding of NRR that may vary for distinct catalytic systems remains to be further explored.

### 2.3 Thermodynamic limits of photocatalytic NRR to NH_3_

Due to the proton-electron transfer of multiple intermediates resulting in sluggish reaction kinetics, the thermodynamic constraints relying on the reaction intermediates and the overall and half-reaction thermodynamics independent of the photocatalysts play the decisive role in the photocatalytic NRR process (Table 1). Despite the overall reaction of NH_3_ production via N_2_ reduction in water (N_2_ (g) +  6H^+^ + 6e^−^  ↔  2NH_3_ (g), *E* ^0^ = +0.55 V vs. NHE) is more thermodynamically feasible than the competing hydrogen evolution reaction (HER) by water reduction, HER is more likely to occur with fewer electrons, decreasing the selectivity toward NH_3_. Besides, the more negative potentials for the intermediate reactions, such as −4.2 V (vs. RHE) for one-electron activation to form surface-bound N_2_ and −3.2 V (vs. RHE) for first proton-coupled one-electron hydrogenation of N_2_ (Zhu et al., 2013; Shi et al., 2020a; Huang et al., 2020), are beyond the photocatalysis capability of most semiconductors without active sites.

**TABLE 1:** Thermodynamic potentials of hydrogenation reactions related to the whole nitrogen fixation pathways.

Reaction equilibrium	*E* ^o^	Ref.	Eqn.
N_2_ (g) + 3H_2_O (l) ↔ 2NH_3_ (g) + 3/2O_2_ (g)	−1.137 V vs. SHE (pH 0)	Hochman et al. (2020)	1
2H^+^ + 2e^–^ ↔ H_2_ (g)	0 V vs. SHE (pH 0)	Shi et al. (2020a)	2
3H_2_O (l) ↔ 3/2O_2_ (g) + 6H^+^ + 6e^–^	+1.23 V vs. NHE (pH 0)	Hochman et al. (2020)	3
N_2_ (g) + 6H^+^ + 6e^–^ ↔ 2NH_3_ (aq)	+0.092 V vs. RHE	Lindley et al. (2016)	4
N_2_ (g) + 6e^–^ ↔ N_2_^−^ (aq)	−4.16 V vs. NHE or −3.37 V vs. RHE (pH 14)	Shi et al. (2020b)	5
N_2_ (g) + 6H^+^ + 6e^–^ ↔ 2NH_3_ (g)	+0.55 V vs. NHE (pH 0)	Bazhenova and Shilov, (1995)	6
N_2_ + 8H^+^ + 8e^−^ ↔ 2NH_4_^+^	+0.27 V vs. NHE (pH 0)	Chen et al. (2018)	7
N_2_ (g) + H^+^ + e^–^ ↔ N_2_H	–3.20 V vs. RHE	Shi et al. (2020a)	8
N_2_ + 2H^+^ + 2e^–^ ↔ N_2_H_2_ (g)	–1.10 vs. RHE	Shi et al. (2020a)	9
N_2_ + 4H^+^ + 4e^–^ ↔ N_2_H_4_ (g)	–0.36 V vs. RHE	Shi et al. (2020a)	10
N_2_H_2_ (g) + 2H^+^ + 2e^–^ ↔ N_2_H_4_ (aq)	0.529 vs. RHE	Fu et al. (2022)	12
N_2_H_4_ (aq) + 2H^+^ + 2e^–^ ↔ 2NH_3_ (aq)	0.939 vs. RHE	Fu et al. (2022)	13
N_2_ (g) + 6H_2_O ↔ 2NO_3_^−^ + 12H^+^ + 10e^–^	+1.24 V vs. NHE	Yifu Chen et al. (2020)	14

The initial chemisorption and activation of N_2_ could trigger the formation of different kinds of species in the subsequent reactions owing to multiple complicated electrons transferred hydrogenation procedures and the presence of reactive oxygen species by water oxidation half-reaction, such as oxygen, hydroxyl radicals ·OH (2H_2_O+ 4h^+^ → O_2_ + 4H^+^, *E* ^0^ = 0.81 V vs. NHE at pH 7 or H_2_O+ h^+^ → OH + H^+^, *E* ^0^ = 2.32 V vs. NHE at pH 7) (Sun et al., 2018). Adversely, the formed O_2_ further captures electrons in the CB to suppress the NH_3_ photosynthesis (Shiraishi et al., 2018; Sun et al., 2018), and the strong oxidation agents ·OH can also further generate nitrite or nitrate from the generated NH_3_ by photooxidation, which are detrimental to N_2_ photoreduction to NH_3_. Because of its weaker interaction with the surface O atom caused by vacancies or doping, metal is further less firmly bound to O following the release of one H atom from adsorbed H_2_O* onto the surface O atom, thus two OH* bonded at the neighboring catalytic sites are easily coupled forming peroxide H_2_O_2_* preferentially adsorbed at active sites, thereafter poisoning the photocatalyst (Liu et al., 2012). The oxidation half-reaction products also oxidize the photogenerated NH_3_ to HNO_3_, leading to a decrease in the NH_3_ yield. Yang et al. (2021) transformed the N_2_ disproportionation reaction into a complete reductive nitrogen photofixation by introducing Au nanoparticles into Fe-TiO_2_ to effectively decompose H_2_O_2_. Some neglected N_2_ fixation products (e.g., N_2_H_4_, NO_2_^−^, and NO_3_^−^, etc.) as essential chemicals should be noted (Table 1, Eqs. 10 and 14), in which N_2_H_4_ as a by-product from afore-mentioned associative alternating pathways has also been detected in the same research (Schrauzer and Guth, 2002; Xiao-Fei Li et al., 2016). Li et al. (Hao Li et al., 2016) discovered that N_2_H_4_ was quickly generated over the BiOCl nanosheets exposed with (010) facets and then gradually disappeared to produce NH_3_, which strongly supports the alternative mechanism. In terms of thermodynamic potentials (Table 1, Eqs. 9 and 12), N_2_H_4_ is more prone to be transformed to NH_3_ from surface–N_2_H_4_ intermediates due to its much weaker N–N single bond. Similar to the hydrogenation pathway to NH_3_, dissociated adsorbed N_2_ molecule is oxidized to the intermediate of metastable NO* by photogenerated h^+^, followed by further oxidation with O_2_ and H_2_O from the reaction media, by which N_2_ is converted to nitrate and nitrite through continuous photooxidation reactions (Comer and Medford, 2018). The previously reported N_2_ photoreduction products are either NH_4_^+^ or NO_3_^−^ (Liu et al., 2019; Dong Zhang et al., 2022), simultaneous coproduction of NH_4_^+^ and NO_3_^−^ through simultaneous reduction and oxidation of N_2_ in pure water, which was demonstrated to occur spontaneously in aqueous solutions (Ren et al., 2020). Another critical aspect in NH_3_ production via photochemical N_2_ reduction is that some key reaction intermediates are instrumental in NH_3_ conversion. Zhao et al. (2022) established a redox pathway with a lower kinetic barrier for NH_3_ photosynthesis, in which N_2_ and O_2_ can be trapped at the oxygen vacancies in ultrathin two-dimensional (2D) CuCo metal-organic frameworks (MOFs) to generate *NO and further be reduced to NH_3_ by visible light. Although the desired NH_3_ conversion and selectivity are swayed by these competing reactions, some by-products also play imperative roles in industrial production and living needs.

The addition of sacrificial agents (typically electron donors such as sulfites, amines, humic acid, ascorbic acid, and alcohols) with oxidation potentials lower than water appeases the requirement for oxidizing ability in some SC photocatalysts, further suppressing electron-hole pair recombination (Shen et al., 2020). On the other hand, the overall production rate of NH_3_ is kinetically balanced by the hole consumption rate on the photocatalyst since electrons and holes are generated in pairs under illumination. Organic alcohols with an α-H adjacent to the OH group(s)-to wit, methanol, ethanol, 2-propanol, ethylene glycol, and so forth, can react with holes in VB to accelerate the production of electrons and liberation of protons (Chen et al., 2015). Among them, methanol is demonstrated to be more appropriate and efficient than other hole sacrificial reagents. Methanol not only loses electrons more easily to consume the accumulated holes due to its lower HOMO (Zhao et al., 2015), but also promotes the solubility of N_2_, which could act as a proton donor and partial electron donor for subsequent reduction reactions (Li et al., 2018). However, methanol as the sacrificial agent could be oxidized to form carbonyl-containing compounds (e.g., aldehyde or ketone) and finally CO_2_, which might interfere with product detection and quantification. The N_2_ fixation reaction can also be facilitated by *CO_2_^−^ produced from the oxidation process of methanol (Swain et al., 2020). Cao and co-workers (Cao et al., 2018) found that the·CO_2_^−^ intermediates transformed from methanol or absorbed CO_2_ affected the nitrogen fixation due to their strongly reducing ability (*E*_CO2/·CO2−_ = 1.8 V) (5N_2_ + 2CO_2_^−^ + 4H_2_O → 2NH_3_ + 2CO_2_ + 2OH^−^) (Dimitrijevic et al., 2011). It should be noted that the NH_3_ formation mechanism in such sacrificial systems should be taken with great caution, the target scavenger should be chosen carefully.

### 2.4 Protocols, evaluation, and detection methods in photocatalytic NRR

Despite the great strides toward photon-driven ammonia production that have been taken, unified rules and standards in this fledgling field should be formulated to ensure the authority and accuracy, by which the impetus imparted forcefully contribute to advance of the sustainable technology. Since the low NH_3_ yield (ppb/ppm level) and ubiquitous contaminants plague experimental practices (Gao et al., 2018; Tang and Qiao, 2019; Zhao et al., 2019), establishing a uniform protocol for rigorous experiments preceding the detection and quantification of NH_3_ are noncontroversial. Therefore, to clarify the source of NH_3_ and ensure data reproducibility, various contamination sources from the environment, latex gloves, human respiration, stable deionized water, photochemical reactor, or the used feed gas, even NH_3_ or amine groups unintentionally introduced by catalysts, should be ruled out to avoid overestimation of the NH_3_ concentration (Bai et al., 2017; Zhao et al., 2021). The photocatalysts, photoreaction set-up and all its components as well as sample tubing should be thoroughly rinsed with fresh ultrapure water and properly stored. Particular emphasis should be put on the NO_*x*_ contaminants derived from N-containing chemicals, protic solvents, or supply gas, which are easily reduced to NH_3_ (Xue Chen et al., 2020; Li et al., 2022a; Feng Wang et al., 2022; Xu et al., 2022). To account for the reliability and repeatability of photoactivity, it is strongly recommended to use reported rigorous experimental protocols (Andersen et al., 2019; Tang and Qiao, 2019; Choi et al., 2020; Shen et al., 2021), which clearly list experimental methods, gas purification, blank and control experiments, especially the isotopic labeling experiments. The ^14^N_2_ and ^15^N_2_ feed gases should be pre-purified to remove any possible NO_*x*_ or NH_3_ to eliminate uncertainty and even false positives of catalytic data (Zhao et al., 2020; Hui et al., 2022; Yuting Wang et al., 2022).

However, controversy exists as to how to obtain reliable detection and quantification of NH_3_ and NH_4_^+^, which severely hampered the growth of this field. Fortunately, the development of measuring techniques further pushed the advance of NH_3_ evaluation. Currently, detection and quantification of NH_3_ could be mainly divided into five methods, including spectrophotometric/colorimetric assays using indophenol blue or Nessler’s reagent, ion chromatography (IC), ion-selective electrode (ISE), fluorescence, and ^1^H-NMR spectroscopy methods (Gao et al., 2018; Zhao et al., 2019). These methods are methodologically sound and get concordant precise results for NH_3_ determination in water systems (Mansingh et al., 2021). However, each of these methods has both advantages and limits for measuring NH_3_. Colorimetric assays are widely available with benefits of good sensitivity, fine accuracy (0–500 µg_NH3_ L^−1^), and low cost (Zhao et al., 2019). The pH, solvent used, presence of certain metal ions, sacrificial agents and their oxides, and nitrogen-containing chemicals can all adversely increase the amount of NH_3_ detected by the coloration methods (Gao et al., 2018). IC is recommended for NH_3_ quantification given its reproducible, precise results with a wider range of NH_3_ estimation, superior efficiency and selectivity, and good stability, but it still suffers from the disadvantage that certain sacrificial agents affect the separation efficiency of the cation-exchange column. NMR spectroscopy, mass spectroscopy, and enzyme assays are also employed as supplementary measures to eliminate the possible false-positive results, the first of which, even quantitatively, could not prove the origin of all ammonia generated. For consistency and scientific rigor, the concentration of NH_3_ detected should be cross-checked with two or more different quantitative methods, even conducted in *in-situ* and continuous monitoring processes for reliable evidences from an unimpeachable source.

**FIGURE 4:**
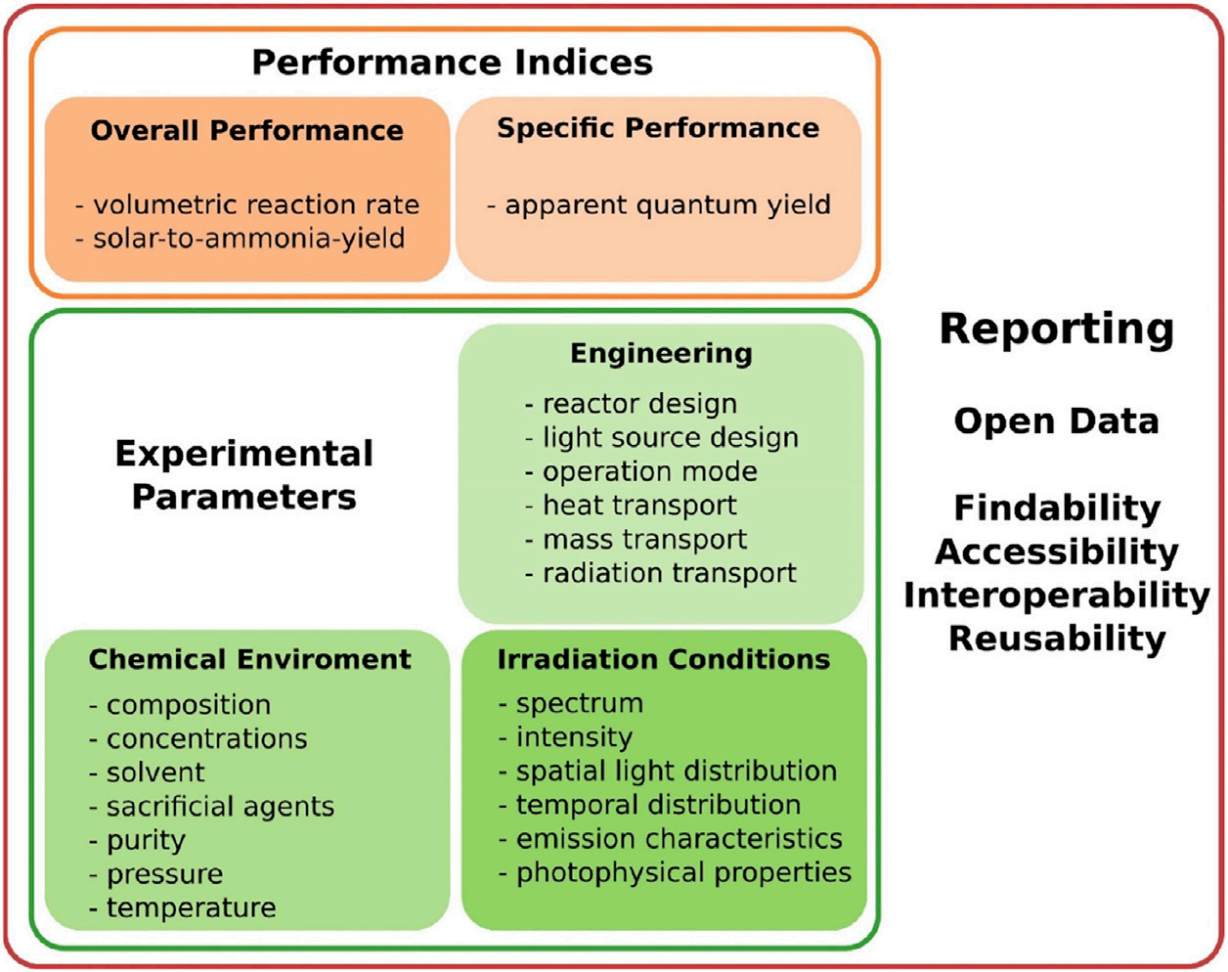
Guidelines for experiment normalization to rule out the impact of various parameters that influence the overall and specific performance indices. Reproduced from ref. (Ziegenbalg et al., 2021). With permission from the John Wiley and Sons.

Given the lack of the standard photocatalytic reaction systems used in conventional laboratory tests (Ziegenbalg et al., 2021), it is therefore necessary to pay particular attention to various vital details during the assessment of photocatalytic nitrogen fixation. Figure 4 presents the effect of important experimental parameters on the observed catalytic performance for photo-driven NH_3_ production. An objective and meaningful comparison of photocatalytic NH_3_ synthesis performance among different groups is heavily reliant on a set of standard experimental conditions (e.g., light source intensity, irradiation wavelength range, photocatalyst dosage, reaction solution volume, reactor type, reaction temperature and pressure, *etc*.). Many technical aspects especially the light source are often missing or not stated in sufficient details**.** Standardization of reactor design is desirable, thereby minimizing the impact of geometry. Reported performance evaluation is primarily based on NH_3_ yield, apparent quantum yield/efficiency (AQY/AQE) and turnover frequency (TOF). However, mass-based performance metrics (*e.g.*, μmol h^−1^ g^−1^) are insufficient because photocatalytic activity is not necessarily proportional to the catalyst mass (Kramm et al., 2019; Huimin Liu et al., 2021). Similarly, AQY/AQE is often used to evaluate the photocatalytic activity under monochromatic light excitation of the same specific wavelength, which is closely related to the wavelength of incident photons and the intrinsic properties of the materials, since photocatalysts generally behave differently on absorption coefficients and photocatalytic activities at each irradiation wavelength (Yuhua Wang et al., 2019). TOF differs as well in terms of the active centers. Solar-to-NH_3_-yield (SAY)/solar-to-NH_3_ (STA) efficiency can be universally used as the practical standard for comparison, which is determined by a solar simulator (AM 1.5G) with an irradiance of 100 mW cm^−2^. With more regard to normalizing evaluation systems, solar-to-NH_3_ energy conversion (SEC) efficiency is suggested to objectively compare the catalytic activity of different materials and assay the future industrialization opportunities (targeted SEC ≈10%) (Rong Zhang et al., 2019; Ziegenbalg et al., 2021).

## 3 Tungsten-based and related photocatalysts for NRR

### 3.1 Metal oxide-based photocatalysts

#### 3.1.1 Tungsten oxides

**FIGURE 5:**
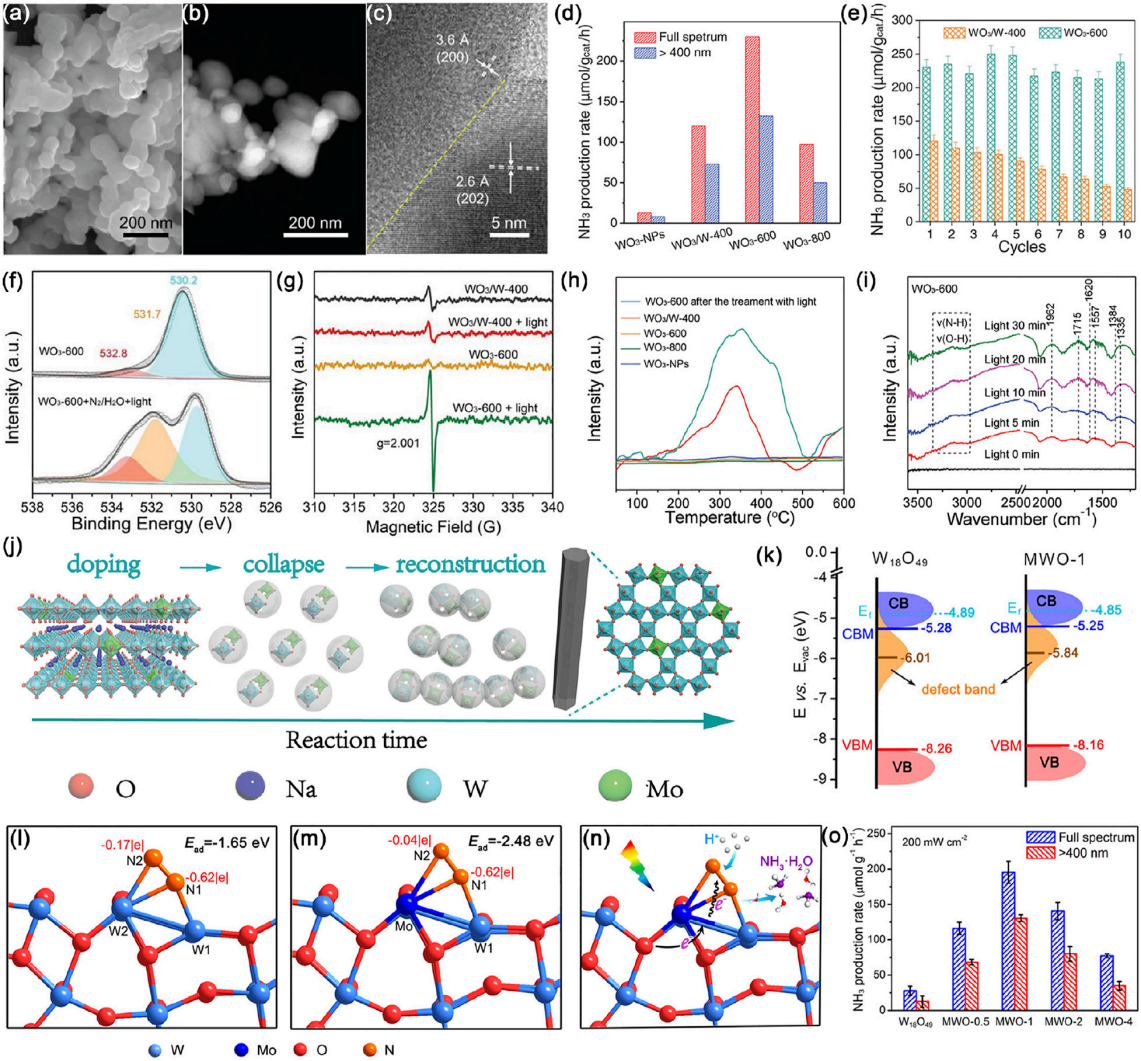
**(A)** Scanning electron microscopy (SEM) image, **(B)** high-angle annular dark-field scanning transmission electron microscopy (HAADF-STEM) image, and **(C)** high-resolution TEM (HRTEM) image of WO_3_-600. **(D)** Photocatalytic NH_3_ production rates over WO_3_-NPs, WO_3_/W-400, WO_3_-600, and WO_3_-800. **(E)** NH_3_ production rates for WO_3_-600 and WO_3_/W-400 over the course of ten rounds of successive reactions. **(F)** Quasi *in situ* XPS spectra of O 1s in WO_3_-600 before and after treatment. **(G)** *In situ* ESR spectra of WO_3_/W-400 and WO_3_-600 before and after light irradiation. **(H)** N_2_-TPD profiles of WO_3_/W-400, WO_3_-600, WO_3_-800, WO_3_-NPs, and WO_3_-600 after the treatment with light. **(I)** *In situ* DRIFT spectra recorded during the photocatalytic N_2_ fixation over WO_3_-600. Reproduced from Hou et al. (2019) with permission from the John Wiley and Sons. **(J)** Schematic illustration of the generation of Mo doped h-WO_3_ crystalline nanowires. Reproduced from Mao et al. (2020) with permission from the John Wiley and Sons. **(K)** Schematic illustration of the electronic band structures of W_18_O_49_ and 1 mol% Mo-doped W_18_O_49_ ultrathin nanowires. Optimized adsorption configurations of N_2_ molecules and their corresponding charge distribution on the surface of **(L)** W_18_O_49_ and **(M)** Mo-doped W_18_O_49_. **(N)** Scheme for photocatalytic N_2_ reduction over Mo-doped W_18_O_49_. **(O)** Photocatalytic ammonia production rates by W_18_O_49_, MWO-0.5, MWO-1, MWO-2, and MWO-4 UTNWs in the first 2 h. Reproduced from Ning Zhang et al. (2018) with permission from American Chemical Society.

Tungsten oxides, commonly denoted as WO_3−*x*_∙*n*H_2_O (*x* < 1, *n* = 0–2), are somewhat distorted in their crystal structure made up of perovskite units, rather not perfect octahedral, with bandgap energies ranging from 2.4 to 2.8 eV depending on their stoichiometries, crystalline structure and density of defects (Zheng et al., 2011; Sun et al., 2019). Tungsten trioxide (WO_3_) is the most popular semiconductor among all tungsten oxides because of its exceptional chromic properties and mixed polymorphs, which manifest in rich and diverse structures (Girish Kumar and Koteswara Rao, 2015; Yin and Asakura, 2019; Cheng and Zhang, 2020). Among different structures of WO_3_, the monoclinic phase is a preferred photocatalyst with the most relatively thermodynamically stable configuration than orthorhombic and hexagonal phases, while triclinic and cubic crystal structures rarely get attention (Zheng et al., 2011; Girish Kumar and Koteswara Rao, 2015; Fan et al., 2021). Hou *et al.* (Hou et al., 2019) developed a facile method to prepare monoclinic WO_3_ via thermal treatment of nanoporous metals, wherein nanoporous WO_3_-600 was composed of connected grains rather than a single grain, containing abundant grain boundaries (GBs) (Figures 5A–C). Impressively, WO_3_-600 showed excellent performance for photocatalytic NRR with an NH_3_ yield rate as high as 230 µmol g_cat._^−1^ h^−1^ without any sacrificial agents at room temperature, 17 times higher than that for WO_3_ nanoparticles (WO_3_-NPs) without GBs (Figure 5D). Moreover, almost 100% of initial activity was maintained even after ten successive reaction rounds over WO_3_-600 (Figure 5E). Quasi *in situ* XPS and *in situ* electron spin resonance (ESR) measurements have been employed to verify the pivotal role of GBs in inducing a large number of operando OVs under light irradiation (Figures 5F,G). These operando OVs served as highly active sites for efficient adsorption and activation of N_2_, which have been confirmed by temperature-programmed desorption of N_2_ (N_2_-TPD) (Figure 5H), directly contributing to easier delivery of photoexcited electrons to adsorbates through metal-oxygen covalency. Then N_2_H* intermediates coupled with protons were observed by *in situ* diffuse reflectance infrared Fourier transform (DRIFT) (Figure 5I). As shown in Figure 5J, sub-5-nm-sized nanowires of hexagonal tungsten oxide (*h*-W O _3_) via a dopant replacement-driven molten salt method also turned out to be excellent photocatalysts with a high NH_3_ production rate of 370 μmol g^−1^ h^−1^, benefiting from unique features of the Mo-doped ultrathin hexagonal structure, thus facilitating carrier separation and dissociation of N_2_ molecules (Mao et al., 2020). Tailoring the morphology of WO_3_ is not only a rational route to investigate the relationship between the microstructure and photocatalytic performances, but also a feasible approach for fabricating highly photoactive nanomaterials.

Since the lattice of WO_3_ withstands a considerable loss of oxygen content, the resulting nonstoichiometric tungsten suboxides (WO_3–*x*_) compositions such as W_20_O_58_, W_18_O_49_ and W_24_O_68_, have suitable bandgap energy, and tunable electronic band structure, charge redistribution, as well as existence of mixed-valence W ions benefiting different degrees of oxygen deficiency in their structures (Song et al., 2015; Sun et al., 2019; Yin and Asakura, 2019; Meng Yang et al., 2020). To accommodate the large energy band of N_2_, Zhang et al. (Congmin Zhang et al., 2018) used a solvothermal method to fabricate defect-rich W_18_O_49_ ultrathin nanowires doped with Mo, where Mo dopants shifted defect-band center up toward the Fermi level (*E*_F_), thereby harvesting more photon energy to provide adequate energetic electrons for N_2_ reduction (Figure 5K). Moreover, the Mo–O covalent bond facilitated the separation and transfer of photogenerated charges from coordinatively unsaturated Mo sites to N_2_ adsorbates, while the formation of the Mo−W bond can effectively enhance the molecular polarization of chemisorbed N_2_ as a reactive dual-active center, resulting in better activation (Figures 5L, N). The as-prepared 1 mol% Mo-doped W_18_O_49_ sample showed enhanced photocatalytic performance with an NH_3_ generation rate of 195.5 μmol g^−1^ h^−1^ and STA efficiency of 0.028% under simulated sunlight **(** Figure 5O **)**. Analogously, Mn^2+^ ions were introduced to replace W sites in the W_18_O_49_ lattice, which not only acted as chemisorption and activation centers for N_2_ and H_2_O molecules, but also facilitated the separation and migration of photogenerated charges (Ying et al., 2019). Tailoring surface oxygen vacancies and doping of tungsten oxides with heteroatoms are effective strategies to increase the number of active sites for N_2_ chemisorption (Mingli Zhang et al., 2020).

#### 3.1.2 Molybdenum trioxides

**FIGURE 6:**
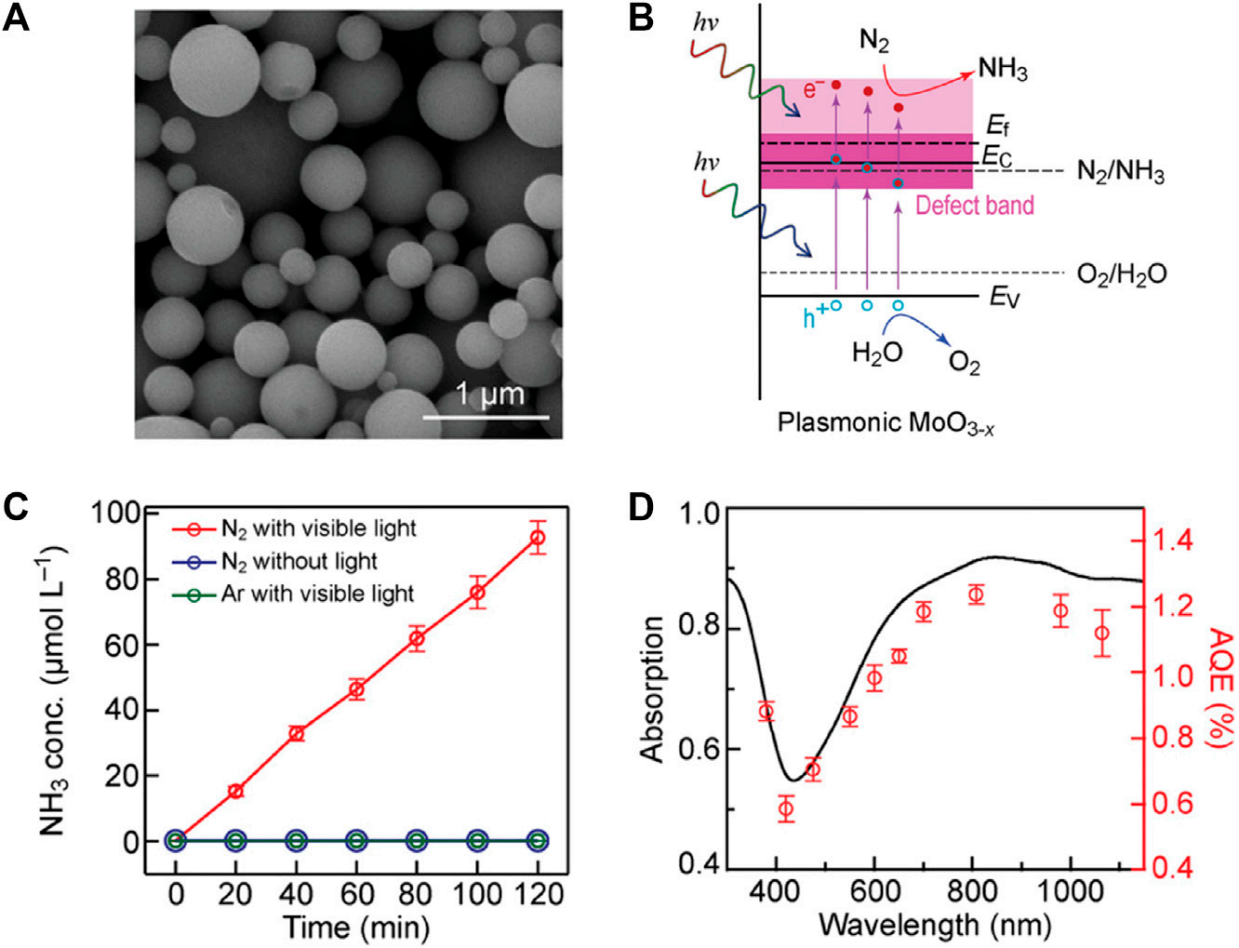
**(A)** SEM image of the MoO_3−x_ spheres prepared at 350°C. **(B)** Schematics illustrating the band structures of the plasmonic MoO_3−x_ photocatalyst. **(C)** Photocatalytic NH_3_ production under different conditions and **(D)** AQE at different wavelengths over the MoO_3−x_ spheres. The light absorption spectrum is plotted for comparison. Reproduced from Bai et al. (2022) with permission from the John Wiley and Sons.

Molybdenum in the same transition metal group with tungsten shares some similar chemical properties, where a non-stoichiometric form MoO_3–*x*_, analogous to WO_3–*x*_, exhibits prodigious potential for solar-driven photocatalysis due to its strong OVs-induced localized surface plasmon resonance (LSPR) absorption in visible-near infrared (vis-NIR) region (Yehuan Li et al., 2019; Zheng Wang et al., 2019; Qiu et al., 2021; Li et al., 2022b). Bai *et al.* (Bai et al., 2022) synthesized Schottky-barrier-free MoO_3−*x*_ spheres via a facile aerosol-spray method for plasmon-driven photochemical N_2_ fixation to NH_3_, which features metal-like free charge carriers with the Fermi level above the bottom of the defect band and the defect band located closely to the conduction band (Figures 6A,B). The MoO_3−*x*_ spheres treated at 350 °C delivered an NH_3_ production rate of 435.57 μmol h^−1^ g^−1^ in 20 vol% methanol aqueous solution under full-spectrum Xe lamp illumination, with an AQE of 1.24% at 808 nm and 1.12% at 1,064 nm and a STA efficiency of 0.057% in pure water under simulated sunlight (Figures 6C,D). Both the measured AQEs and the corresponding wavelengths under NIR region are among the highest values to date. Specifically, the OVs enable a perfect functional combination of rich active sites for N_2_ absorption with broad-spectrum plasmon-induced hot electrons and empty states in the defect band within the MoO_3−*x*_ spheres, which facilitates the multi-electron reduction-oxidation (red-ox) reactions involved in photocatalytic N_2_ reduction. With the Schottky-barrier-free characteristic, the hot electrons moved freely in the defect-induced electronic states and the conduction band reduced those adsorbed and activated N_2_ molecules trapped at the OVs to produce NH_3_, as illustrated in Figure 6B. The defective MoO_3−*x*_ has been exploited as matrix support to valorize rare earth La single-atom catalysts (SACs) via simple Lewis acid-base interactions due to their well-defined surface structure and high degree of anisotropy (Liu et al., 2022). The density functional theory (DFT) calculations revealed that a La single atom theoretically tends to occupy terminal OVs and coordinate with 2-coordinated O site (Mo–O–Mo, O_2c_) to form O_2c_-La-O_2c_ coordination. Moreover, the role of La atoms on O_2c_–La–O_2c_ site was further clarified, which pumps energetic electrons from their unsaturated 5*d* orbitals into the π * 2*p* orbital of the adsorbed N_2_, boosting N_2_ adsorption and activation. Isolated atomically dispersed La atoms anchored on MoO_3−*x*_ support were observed by the aberration-corrected HAADF-STEM and corresponding rainbow-colored images. The O_2c_−La−O_2c_ configuration close to two oxygen coordination environment was further verified by X-ray absorption fine structure (XAFS), by which La−La bond cannot be distinctly observed in La/MoO_3−*x*_. La/MoO_3−*x*_ possess an NH_3_ production rate of 209.0 μmol h^−1^ g^−1^ without any hole scavenger under visible light, nearly 10 times that of the support. Enhanced adsorption of nitrogen and the symmetric alternative pathway following a side-on bridging adsorption configuration have been corroborated by *in situ* FT-IR spectra with the combination of DFT calculations, thus La single atoms substantially amplify the activation of N_2_ toward successive hydrogenation, while lowering the formation energy barrier for *NNH → *NHNH process.

#### 3.1.3 Mixed valence cobalt oxides

Cobalt, one of the earth-abundant first row transition metals, has gained tremendous attention for photocatalytic N_2_ reduction and conversion due to its eligibility for N_2_ dissociation and tunable activity (Chu et al., 2019; Gao et al., 2019), whereas its oxides with mixed-valence states of Co species have poor photostability due to photo-corrosion arising from half oxidation reaction. Combination of CoO_x_ with 2D carbon material supports have been rationally explored to optimize their electronic structures and tailor the active site density (Ahmed et al., 2019; Chu et al., 2019; Zhi-Yuan Wang et al., 2020; Lu et al., 2022). For example, Liu *et al.* (Yuxin Liu et al., 2021) fabricated CoO_*x*_ quantum dots anchored on porous graphdiyne (GDY) (GDY@CoO_*x*_QD) to construct highly efficient and robust catalysts via a facile *in-situ* growth strategy for photocatalytic NRR. The composite photocatalysts featured with superlative activity and stability under various conditions as a result of strong quantum effect and a highly compatible synergistic effect. The three-dimensional configurations of the self-supported GDY@CoO_*x*_QD nanosheet array and uniformly dispersed CoO_*x*_QD on the porous GDY surface were confirmed by SEM and HRTEM, respectively. An average NH_3_ yield rate of 46 independent experiments over the GDY@CoO_*x*_QD in 0.1 M Na_2_SO_4_ aqueous solution (pH 7) up to 19,583 μmol g^−1^ h^−1^ was attained, exceeding most reported catalysts. Equally importantly, the six different batches exhibited long-term stability of 10 h with nearly constant NH_3_ yield rates. By comparing the Co XPS results of CoO_*x*_QDs on different supported carbon materials and the XANES of GDY@CoO_*x*_QD before and after the reaction, it was concluded that the mixed-valence states of Co (Co^3+^ and Co^2+^) played a pivotal role in enhancing the reaction activity. As revealed by the DFT calculations, the introduction of GDY and the coexistence of Co^2+^/Co^3+^ could facilitate the electron transfer to form a strong *d*-π* (unocc) antibonding orbital interaction above *E*_F_ and *d*-π* (occ) bonding orbital interaction below *E*_F_. This is beneficial for weakening the bond order and bond strength of N≡N bond.

Lu et al. (2022) also reported that different cobalt oxide species were responsible for innate active properties, resulting in a synergistic effect on the two half reactions with reduction and oxidation spatially separated at CoO and Co_3_O_4_, respectively. The CoO-Co_3_O_4_ mixed-oxide (CoO dominated) composites on reduced graphene oxide (RGO) manifested a remarkable NH_3_ formation efficiency of 89.1 μmol g^−1^ h^−1^, over 14 times that of each single component. Furthermore, the photoreaction-induced cation oxidation (CoO to Co_3_O_4_) was reduceable/recyclable by photo-reactivating the non-active Co_3_O_4_ back to the active CoO at room temperature, thus leading to well-maintained NRR activity after six cycles of operation. As indicated by XANES, XPS, and HRTEM measurements, the compositions were completely transformed into Co_3_O_4_ during the 8 h of NRR and converted back to dominant CoO after the reactivation. The component CoO in the composite entailed deep-red-light absorbing defect states, which hindered carrier recombination. The band structure of CoO/Co_3_O_4_ formed a direct Z-scheme heterojunction, in which the electrons at the CB of CoO reduced N_2_ molecules and the holes at the VB and defect energy levels of Co_3_O_4_ oxidized H_2_O molecules.

#### 3.1.4 Other transition metal oxides

**FIGURE 7:**
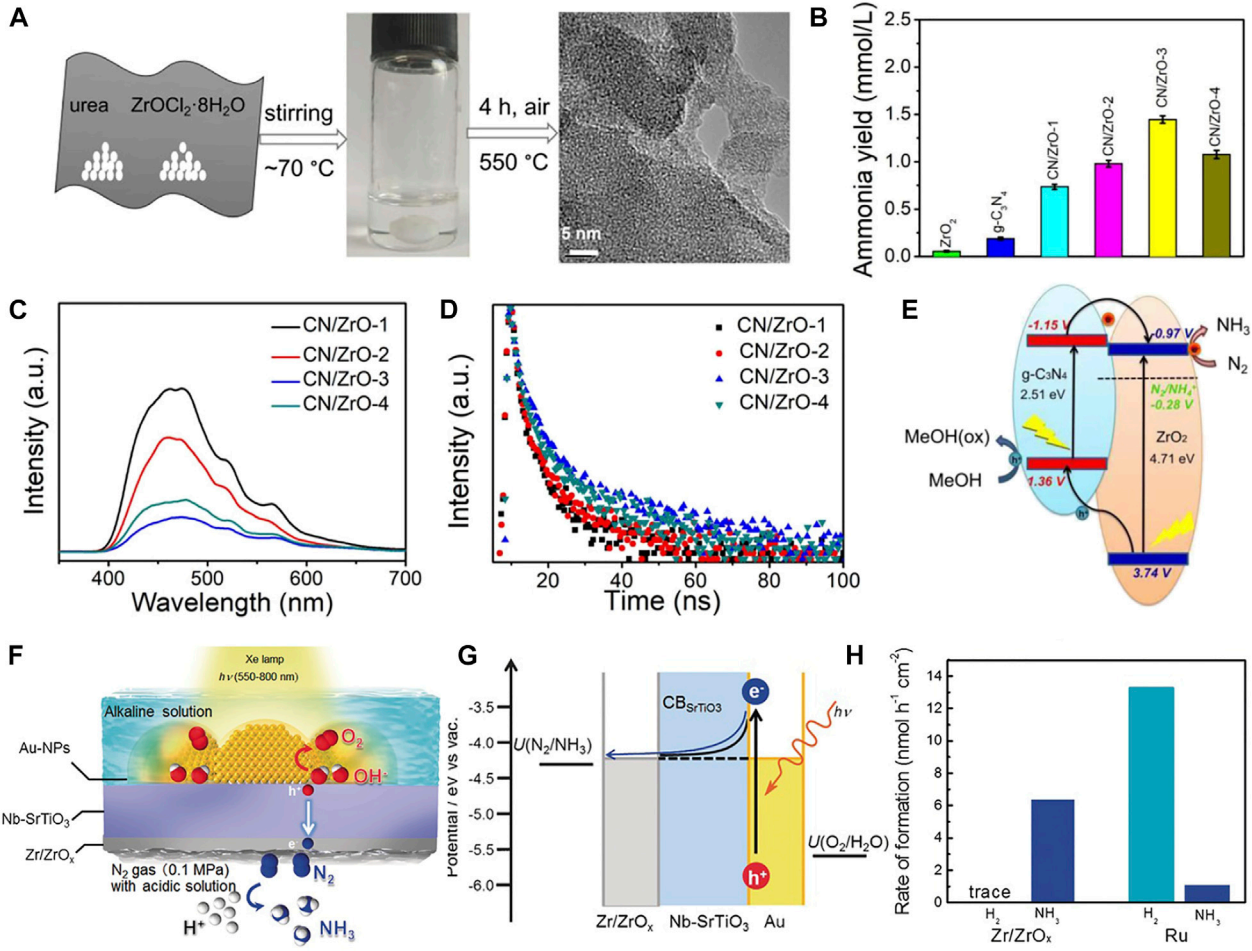
**(A)** Schematic of *g*-C_3_N_4_/ZrO_2_ composite synthesis. **(B)** NH_3_ yields of pure ZrO_2_, g-C_3_N_4_ and *g*-C_3_N_4_/ZrO_2_ composites with different ZrO_2_ contents. **(C)** Photoluminescence (PL) spectroscopy and **(D)** time-resolved PL (TRPL) decay spectra. **(E)** Schematic of mechanism for photocatalytic N_2_ fixation over *g*-C_3_N_4_/ZrO_2_ composites. Reproduced from Mou et al. (2019) with permission from American Chemical Society. **(F)** Schematic of NRR on Au-NPs/Nb-SrTiO_3_/Zr/ZrO_*x*_ photoelectrode. **(G)** Energy-level diagram of the plasmon-induced NH_3_ synthesis device. *U*: redox potential. **(H)** NH_3_ formation rate over Au-NPs/Nb-SrTiO_3_/Zr/ZrO_*x*_ and Au-NPs/Nb-SrTiO_3_/Ru. Reproduced from Oshikiri et al. (2016) with permission from the John Wiley and Sons.

Non-noble metal oxides have gained tremendous expectations as promising alternative NRR catalysts primarily owing to their high chemical stability, ease of synthesis, and minimization of noble metals consumption (Liang Yang et al., 2020; Gao et al., 2022; Zhen Zhao et al., 2022). Only a few metal oxides such as VO_2_ (Haiguang Zhang et al., 2019), Ta_2_O_5_ (Fu et al., 2019), Nb_2_O_5_ (Han et al., 2018; Kong et al., 2019), NbO_2_ (Huang et al., 2019), and ZrO_2_ (Xu et al., 2020) have been reported for electrocatalytic NRR, However, these metal oxides suffer from large band gap, poor light absorption capacity toward visible light, low quantum efficiency, and fast recombination rate of photogenerated excitons, restricting their applications in photocatalytic NRR. The insulating material ZrO_2_ with a band gap of ∼5.0 eV (Xu and Schoonen, 2000) can absorb ultrahigh UV light (Gaggero et al., 2021). In addition, it possesses high mechanical strength, non-toxicity, and corrosion resistance. Intensive research has been concentrated on engineering and visible photosensitization of high band gap oxides based on Zr elements of the fourth subgroup (Oshikiri et al., 2016; Caiting Feng et al., 2022). Theoretical calculations revealed that the adsorption energy of N (ΔN*) is much lower than that of H on the ZrO_2_ surface in the aqueous photocatalytic NRR process. This suggests that ZrO_2_ preferentially adsorbs N atoms and baffles the reduction of H_2_O to H_2_ (Skúlason et al., 2012; Tao et al., 2019). Mou *et al.* (Mou et al., 2019) demonstrated photocatalytic NRR to NH_3_ based on amorphous ZrO_2_ in association with *g*-C_3_N_4_ as a visible light harvester. The *g*-C_3_N_4_/ZrO_2_ lamellar composites were constructed by a simple one-step pyrolysis of the deep eutectic solvent ZrOCl_2_·8H_2_O/urea (Figure 7A). The composites imparted an optimal NH_3_ yield rate of 1,446 μmol L^−1^ h^−1^ under visible light illumination, noticeably outperforming each individual counterpart (Figure 7B). The introduction of amorphous ZrO_2_ restrained the hydrogen generation and facilitated N_2_ reduction. A synergy between amorphous ZrO_2_ and *g*-C_3_N_4_ was created contributing to the rapid photoproduced electron–hole pair separation and transfer (Figures 7C–E). ^15^N isotope analysis verified the contamination-free N_2_ photofixation in this work. Similarly, Oshikiri and co-workers designed a multi-component photocatalytic system of Au-NPs/Nb-SrTiO_3_/Zr/ZrO_*x*_ *.* The composite was shown to have high affinity to NH_3_. Plasmonic-induced charge separation happened at the Au/SrTiO_3_ interface, thereby allowing occurrence of oxidation reactions on gold nanocrystals and N_2_ reduction on the ZrO_x_@Zr coating (Figures 7F,G). Figure 7H shows the large interface of ZrO_x_@Zr and Nb-SrTiO_3_ affording high selectivity and efficiency for NH_3_ synthesis owing to the advantage of a stronger binding of ZrO_x_@Zr to N atoms relative to H atoms compared to Ru.

### 3.2 Oxometallate-based photocatalysts

#### 3.2.1 Bismuth-based oxometallates

Among various polyoxometalates, bismuth-based compounds such as Bi_2_WO_6_, Bi_2_MoO_6_ of Aurivillius structure, and BiVO_4_ of Scheelite structure, are another class of oxides of interest because of their considerable chemical stability, up-shifted VB resulting from hybridization between Bi 6*s* and O 2*p* states and narrow band gaps, together with their similar layered crystal structures complimentary for charge separation and transfer (Zhang et al., 2011; Hao et al., 2020; Wenjie Liu et al., 2020; Utomo et al., 2022). Besides, the hybridization of Bi 6*s* and O 2*p* levels accounts for their largely dispersed VB, benefiting the migration of photoinduced holes and thus improving the oxidative reactions (Huang et al., 2020). Bi_2_WO_6_ consists of accumulated layers of discontinuous [Bi_2_O_2_]^2+^ and octahedral [WO_4_]^2−^ sheets, and Bi_2_MoO_6_ is composed of [MoO_2_]^2+^ layers and [Bi_2_O_2_]^2+^ layers, while BiVO_4_ features with a monoclinic crystal system comprising BiO_8_ and VO_4_ groups (Cooper et al., 2014; Dai et al., 2016; Zhu et al., 2022). Nevertheless, the potentially high NRR activities are profoundly impeded by their intrinsic shortcomings, such as the photo-corrosion susceptibility for Bi_2_WO_6_, the limited light absorption in the ultraviolet region for Bi_2_MoO_6_, the poor water oxidation kinetics and slow mobility of photo-excited charge carriers for BiVO_4_ (Girish Kumar and Koteswara Rao, 2015; Xin Liu et al., 2020). Also, they suffer from limited interaction with nitrogen and weak reducing ability as the CB is not sufficiently negative (Hao et al., 2020). To alleviate these problems, heteroatom doping (Meng et al., 2019; Lin Liu et al., 2021), control of facet exposure (Zhang et al., 2021), defect engineering (Haitao Li et al., 2021; Libo Wang et al., 2021; Cai Feng et al., 2022; Guoan Wang et al., 2022), heterostructure construction (Fei et al., 2019; Shende et al., 2019; Xue et al., 2019; Vesali-Kermani et al., 2020a; Chao Liu et al., 2020; Xuerui Zhang et al., 2022), morphology modification (Ning Zhang et al., 2018; Zhou et al., 2019; Sun et al., 2020; Bao et al., 2021), and regulation of internal electric fields (Lv et al., 2018) have been employed to improve the photocatalytic efficiency of N_2_-to-NH_3_ conversion.

**FIGURE 8:**
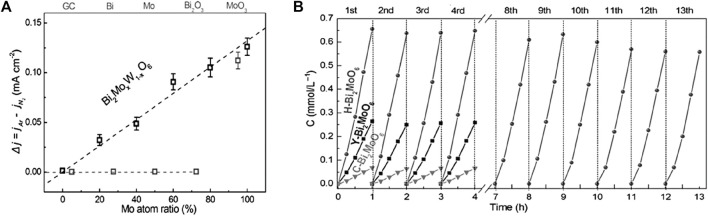
**(A)** The relative current densities (Δ*j* = *j*_Ar_ – *j*_N2_) measured at 0.6 V vs. saturated calomel electrode (SCE) of different samples. **(B)** Multicycle photocatalytic NH_3_ generation from air of different samples under simulated sunlight irradiation. Reproduced from Zhao et al., 2016 with permission from the John Wiley and Sons.

Hao et al. (2016) doped Bi_2_WO_6_ with different ratios of Mo, combined with exposed edge unsaturated Mo atoms as the active center for N_2_ adsorption, activation and photocatalytic reduction (Figure 8A). Benefiting from exposed active sites, narrower bandgap, and ultrasmall subunits in Bi_2_MoO_6_ system (H-Bi_2_MoO_6_), N_2_ molecules from air were transformed into NH_3_ with an NH_3_ evolution rate of 1.3 mmol g^−1^ h^−1^ under simulated sunlight illumination, ≈9.5 times higher than bare Bi_2_MoO_6_ (Figure 8B). In another work, Wang *et al.* demonstrated creation of enriched surface OVs via surface-layer Br doping into Bi_2_MoO_6_ (BMO) which boosted photocatalytic NRR activity owing to enhanced chemisorption of N_2_ molecules, enlarged surface area, improved photogenerated charge separation and transfer efficiencies (Lin Wang et al., 2022). The as-made hierarchical BMO microspheres comprised small nanosheets, in which two Br replaced one MoO_4_^2−^ with OH coordination balancing the crystal structure. DFT simulation indicated that Br doping promoted the formation of surface OVs at adjacent Bi, thus inducing the fabrication of the surface/internal homojunction to enhance the photogenerated charge separation. Addition of methanol as an electron donor (eliminating holes) further accelerated the generation of NH_3_ with a rate of 4.77 μmol h^−1^. The BMO showed no decrease in photocatalytic activity after five consecutive runs, manifesting its high stability, while the low stability of BMO80 in pure water indicates photogenerated hole oxidation induced by Br loss and OV reduction. Exposing different crystal facets provides enhanced spatial separation of photogenerated electrons and holes between different crystal facets. For instance, Zhang *et al.* (Zhang et al., 2021) controlled the growth of (040) and (110) facets of single-crystal BiVO_4_ by adjusting pH, and studied the correlation of facet ratios of BiVO_4_ crystals with photocatalytic NRR performance. They found that the activity was linearly dependent on the ratio of exposed *S*_(040*)*_/*S*_(110)_. Separation of space charges was boosted by *in-situ* photo-deposition of Ag NPs and MnO_*x*_ selectively loaded on the respective (040) and (110) planes. This enabled creation of a built-in electric field (BIEF) between (040)/(110) facets. Based on the results of active sites and DFT calculations, the cycle of oxygen vacancy-V^4+^/V^5+^ in the (040) facets was inferred to be the exact active site for photocatalytic NH_3_ synthesis. V^4+^ was proposed to enhance chemisorption of N_2_ while V^5+^ behaved as an electron transfer bridge, and the photogenerated electrons trapped in OVs provided driving force for NRR.

#### 3.2.2 Polymetallic oxides

Most ternary metal oxides first used for photocatalytic nitrogen fixation are titanate or Bi-based oxide systems (Chen et al., 2018; Huang et al., 2020). Higher conversion efficiency for mingled transition metal composite oxides such as Sb_2_MoO_6_ (Mousavi et al., 2022), SrMoO_4_ (Luo et al., 2019), LaCoO_3_ (Haiguang Zhang et al., 2019), CoFe_2_O_4_ (Zheng et al., 2020), Ni_3_V_2_O_8_ (Vesali-Kermani et al., 2020b), KNbO_3_ (Xing et al., 2019; Xing et al., 2020; Chamack et al., 2022), LiNbO_3_ (Xiazhang Li et al., 2020), have been reported compared to corresponding monometallic oxides. Most mixed metal oxides are perovskite oxide compounds with wide bandgaps (Zejian Wang et al., 2021). To overcome the large band gap issue, exotic element doping, noble metal loading, and coupling with other semiconductors can be applied to broaden the light absorption range. For instance, doping of SrMoO_4_ with Fe simultaneously introduced defect states and Fe–Mo–O active centers acting as the active sites for N_2_ adsorption, which minimized bandgaps to extend the absorption edge (Luo et al., 2019). As the doping concentration increased, the intrinsic bandgap became narrowed, enabling utilization of visible light. However, the excess doped heteroatoms could act as recombination sites for photoproduced charge carriers, as reflected that the normalized photocurrent transient decreased with that of NH_3_ yield at higher doping concentrations.

**FIGURE 9:**
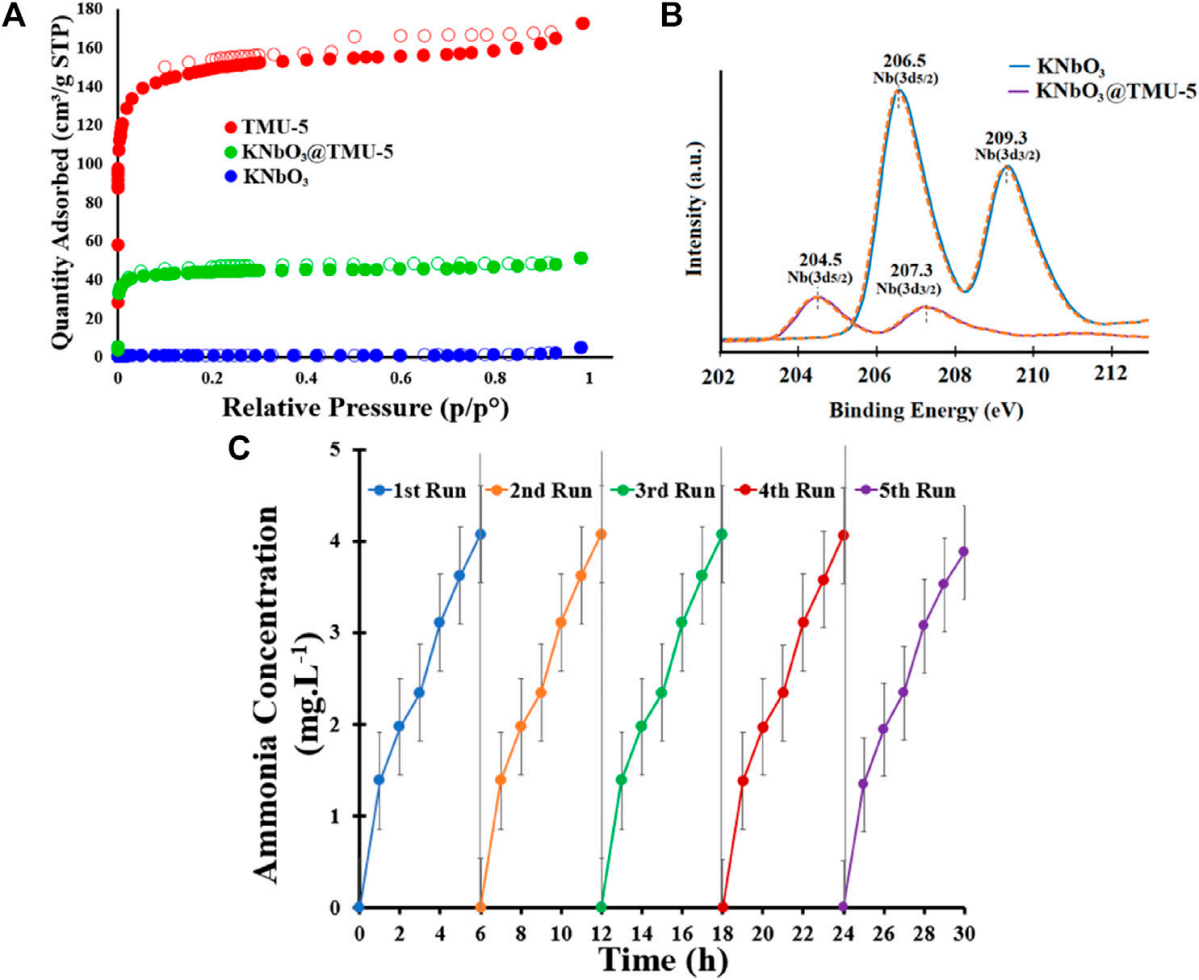
**(A)** N_2_ adsorption/desorption isotherms for the KNbO_3_@TMU-5 composite and its components. **(B)** Core-level XPS scans of Nb atoms of KNbO_3_ and KNbO_3_@TMU-5. **(C)** NH_3_ concentration versus cycling test on KNbO_3_@TMU-5. Reproduced from Chamack et al. (2022) with permission from American Chemical Society.

Niobates have emerged as a research hotspot because of their sufficiently negative CB potential endowing photogenerated electrons with strong reducibility and their spontaneous polarization nature promoting surface charge separation (Chen et al., 2019; Chao Liu et al., 2020; Nunes et al., 2020). Chen *et al.* (2021a) demonstrated the synergy effect between Bi_2_S_3_ and KTa_0.75_Nb_0.25_O_3_ (KTN) under simulated sunlight irradiation and the simultaneous action of light and ultrasonic irradiation. The hybrid displayed high piezo-photocatalytic performance with an NH_3_ production rate reaching 581 μmol L^−1^ g^−1^ h^−1^. All samples depended on KTa_0.75_Nb_0.25_O_3_ to provide the photo/piezogenerated electrons for promoting spatial charge separation, which likely played a dominant role for nitrogen fixation. A pioneering study that coupled conventional KNbO_3_ with photoactive MOFs was conducted by Chamack *et al.* (Chamack et al., 2022). Introduction of ([Zn(OBA) (BPDH)_0.5_]_*n*_·1.5DMF (TMU-5) enhanced the photocatalytic performance owing to the porosity, high surface area, and higher density of negative charges on Nb sites observed by N_2_ adsorption/desorption isotherms and XPS scans, respectively (Figures 9A,B). In addition, KNbO_3_@TMU-5 also exhibited good stability during the five-cycle test (Figure 9C).

#### 3.2.3 Polyoxometalates

**FIGURE 10:**
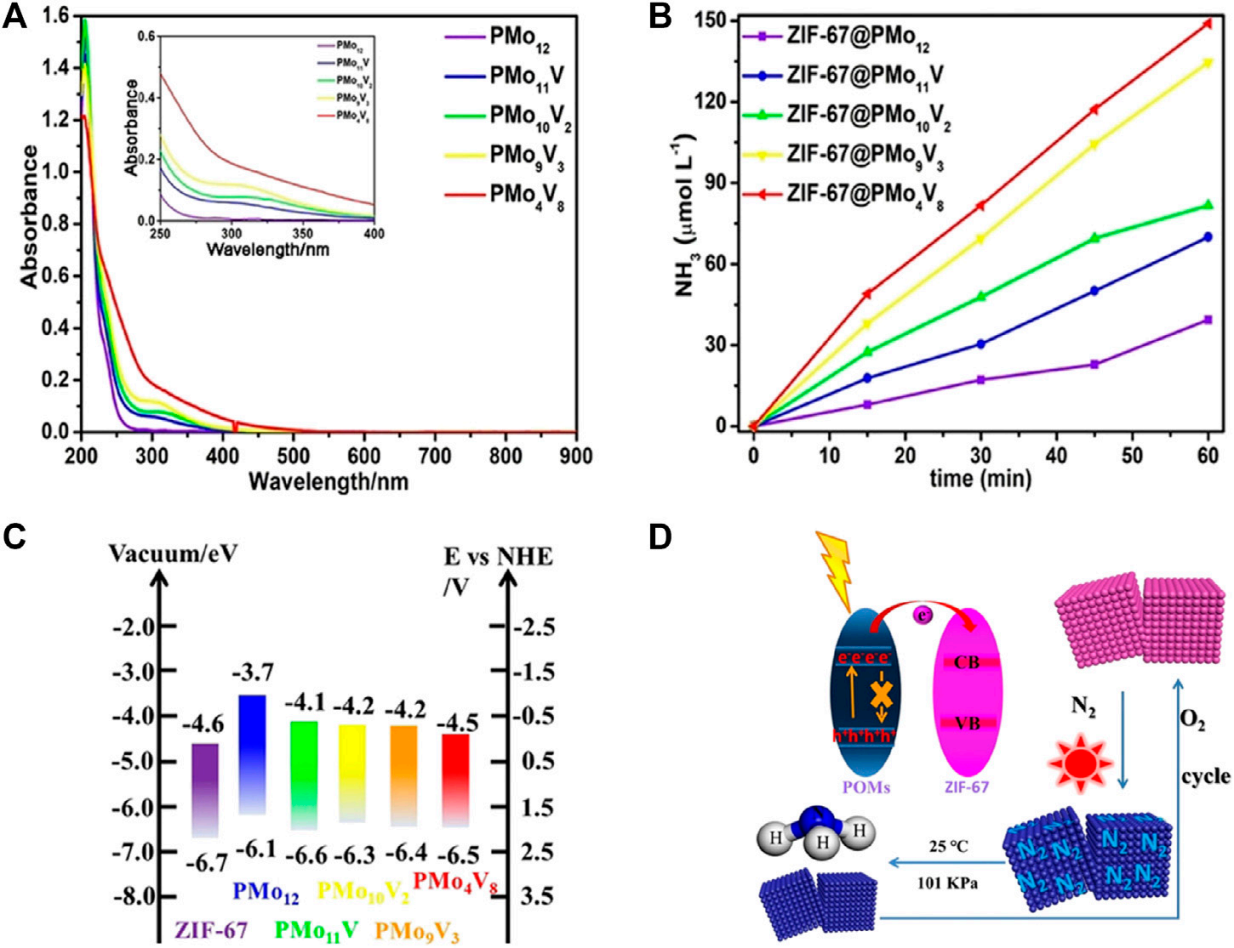
**(A)** UV/Vis absorption spectra of various V-substituted POMs. **(B)** NH_3_ yield as a function of reaction time on various ZIF-67/POMs hybrids. **(C)** Energy levels and **(D)** electron-transfer mechanism for ZIF-67/POMs hybrids. Reproduced from Hongda Li et al. (2020) with permission from the John Wiley and Sons.

Polyoxometalates (POMs) are a class of discrete inorganic polynuclear anionic molecular metal-oxo clusters composed of cations and polyoxometalate polyanions linked together by shared oxygen atoms to form closed 3-dimensional frameworks. Corner-sharing metal oxide polyhedra (MO_*x*_, *x* = 4, 5, and 6) are the basic building blocks, where M usually represents V, Nb, Mo, W, and Ta in high oxidation states (Yin et al., 2018; Gu et al., 2021; Horn et al., 2021). Semiconductor-like POMs or their constituent hybrids are considered as prodigious photocatalytic materials for NRR due to the following reasons: 1) the reversible gain or loss of a specific number of electrons and diverse active sites furnishing the reversible redox capability and modifiable stability; 2) the preponderance of POMs as an electron “reservoir”; 3) the well-defined HOMO–LUMO gap contributing to oxygen (ligand)-to-metal charge transfer; 4) definite particle sizes and dimensions together with the maintenance of structural intactness (Xiao-Hong Li et al., 2019; Xin Wang et al., 2020; Gu et al., 2021; Horn et al., 2021; Yuan Feng et al., 2022). Xiao *et al.* (Xiao et al., 2018) successfully covalently bonded the polyacid cluster [H_4_SiO_40_W_12_] (SiW_12_) to KOH-modified carbonitride graphite nanosheets through a phosphate bridging strategy. This allowed a rapid transfer of photogenerated electrons. The polyacid anion in POMs was suggested to function as an effective binder and electron transport chain, enhancing the interaction with the carrier and electron transport. Cyclic voltammograms of 30-SiW_12_/K-C_3_N_4_ showed that a reversible two-electron redox reaction occurred at −0.06 V and −0.36 V, implying that 30-SiW_12_/K-C_3_N_4_ could act as good containers of electrons and protons to provide protons for the N_2_ reduction. Integration of porous zeolitic imidazolate framework-67 (ZIF-67) (to enhance N_2_ adsorption) with various TM-substituted POMs (PMo_12−x_V_x_, x = 1, 2, 3, 8) (to supply multiple electrons), was investigated by Li *et al.* (Xiao-Hong Li et al., 2020). The light absorption of POMs was intensified to red shift by introducing V into POMs caused by the difference in the number of V hyperchromic effects (Figure 10A). The photocatalytic N_2_ reduction activity increased with increasing the number of V atoms with stronger redox ability than Mo (Figure 10B). The ZIF-67/POM composites attained higher catalytic performance than that of ZIF and POMs alone with the highest N_2_ fixation efficiency reaching 149.0 μmol L^−1^ h^−1^ and a STA efficiency of up to 0.032% for ZIF-67@PMo_4_V_8_. POMs not only improved the utilization of light energy but also easily excited electrons under light conditions to participate in the catalytic process (Figure 10C). Reduced POMs could be regenerated to form oxidized POMs in the presence of oxidants (such as O_2_), enabling a complete self-healing and circulatory system (Figure 10D).

Given the solubility of POM, there are recycling problems with environmental pollution. To overcome this issue, some strategies have been developed to reinforce its stability during NRR, including adhesive (Tianyu Wang et al., 2020), coupling with MOFs (Tianyu Wang et al., 2021), and interfacial interaction (Yuan Feng et al., 2022). For example, Su et al. (2022) fabricated SiW_12_ encapsulated Cr-MOFs (MIL-101(Cr)) hybrids, which were applied to N_2_ photocatalysis affording an NH_3_ yield rate of 75.56 μmol h^−1^ g^−1^, about 10 times that of Na_4_SiW_12_O_40_. SiW_12_ of polyoxometalates was supposed to be the active center, which could be regulated to locate in different cavities of MIL-101(Cr) by controlling the synthesis method. This enhanced the separation efficiency of photoexcited electron-hole pairs. The N_2_ reduction activity and photocatalyst structure did not change much after 5 cycles. The cooperative effect between the porous MIL-101(Cr) and SiW_12_ was hypothesized to boost the nitrogen fixation efficiency.

### 3.3 Transition metal chalcogenides

**FIGURE 11:**
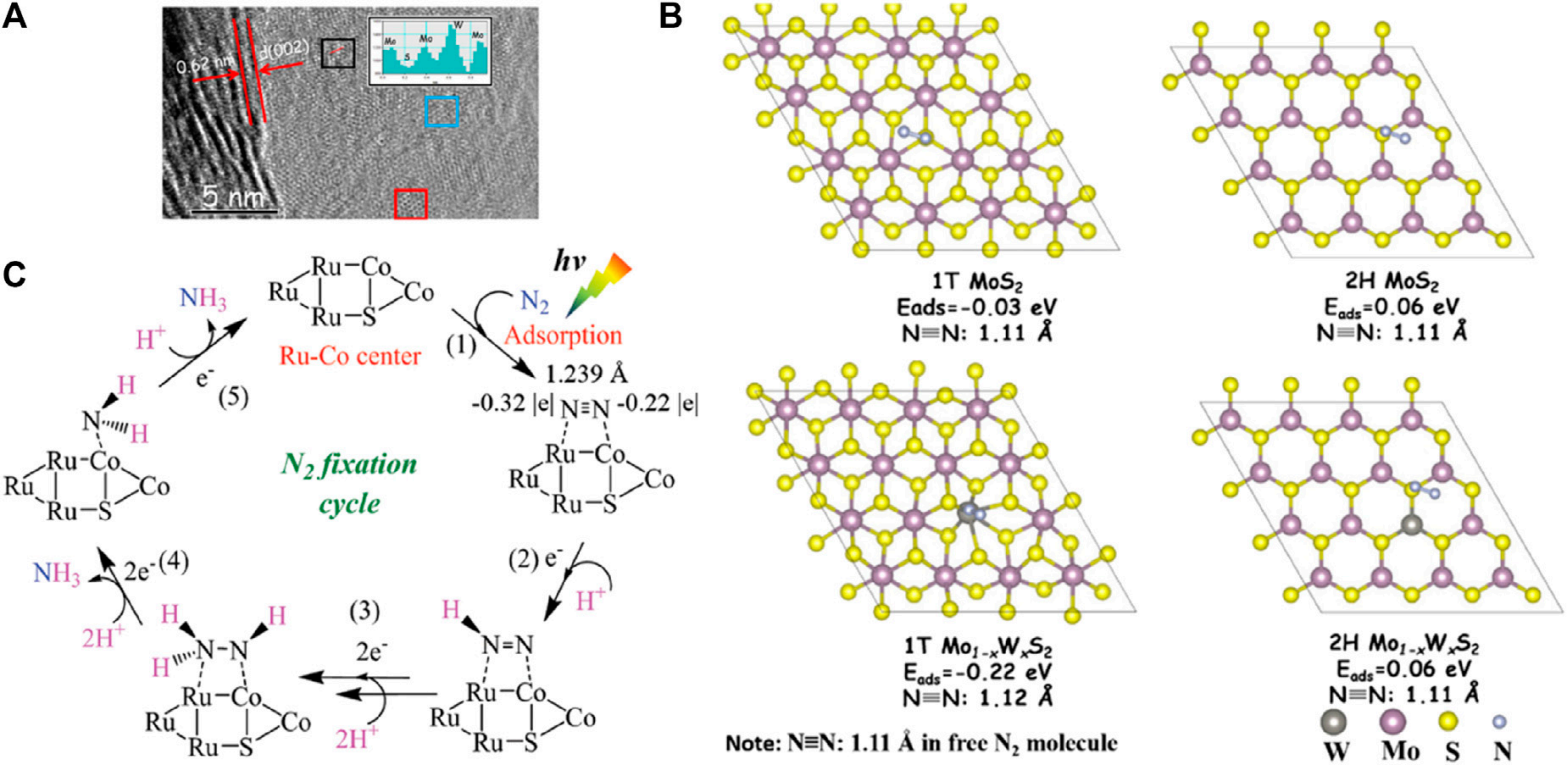
**(A)** HRTEM image of MWS-2 nanosheets. The red and blue squares represent the 2H and 1T structures, respectively; **(B)** Different adsorption energies of N_2_ molecules on 1T MoS_2_, 1T Mo_1−x_W_x_S_2_, 2H MoS_2_, and 2H Mo_1−x_W_x_S_2_. Reproduced from Qin et al. (2021) with permission from American Chemical Society. **(C)** Proposed NRR reaction schemes on Ru-SV-CoS/CN. Reproduced from Yuan et al. (2020) with permission from the John Wiley and Sons.

Transition metal chalcogenides (TMDs) have attracted heightened research interest for photocatalytic NRR primarily owing to their outstanding optical properties (wide spectral response range), relative nontoxicity, liquid media stability, superior electronic mobility, and intrinsic catalytic activity (Sun et al., 2018; Lei et al., 2020; Shen et al., 2020; Shen et al., 2021). Sun et al. (2017) presented the trion-induced NRR on ultrathin sulfur-vacancies (SVs)-rich 2D MoS_2_, where photoexcited electron-hole pairs combined the doping-induced charges to form trions, bound multiple electrons and located around the Mo sites in MoS_2_, which lowered thermodynamic barriers and favored the simultaneous six-electron transfer to produce NH_3_. N_2_ molecules were prone to be absorbed at the SV sites and activated by the electron-rich species to form NH_3_ over the SV-tuned ultrathin MoS_2_. A quasi-stable NH_3_ evolution rate of 325 μmol g^−1^ h^−1^ was attained without using any sacrificial agent or cocatalyst. Qin and coworkers employed a simple one-step hydrothermal method to prepare ultrathin alloyed Mo_1–*x*_W_*x*_S_2_ nanosheets with tunable hexagonal (2H)/trigonal (1T) phase ratios using Na_2_MoO_4_·2H_2_O, Na_2_WO_4_·2H_2_O, and thiourea as Mo, W, and S precursors, respectively. Phase engineering and appropriate W doping markedly boosted the N_2_ photoreduction efficiency. As shown in Figures 11A, the alloys maintained a layer structure during the hydrothermal process, and the 1T, 2H, and polymorph structural domains were also observed in the alloyed Mo_1–*x*_W_*x*_S_2_ nanosheets. The alloyed Mo_1–*x*_W_*x*_S_2_ nanosheets with a 1T phase concentration of 33.6% and Mo/W of 0.68:0.32 (MWS-2) were found to reach the maximal N_2_ fixation rate of about 111 μmol g^−1^ h^−1^ under visible light, 3.7 (or 3)-fold higher than that of pristine MoS_2_ (or WS_2_). DFT calculations revealed that N_2_ had the highest negative adsorption energy on 1T Mo_1–*x*_W_*x*_S_2_ compared to that on 1T MoS_2_, 2H MoS_2_, and 2H Mo_1–*x*_W_*x*_S_2_ (Figure 11B). Meanwhile, *in situ* N_2_ absorption XANES techniques interpreted the energy shift on the peaks of the Mo *K*-edge as the ascending valence states of Mo, and that of W *L*-edge as a higher electron density state in W 5*d* orbitals, resulting in the migration of many electrons from Mo to W. Based on theoretical calculations and photochemical experiments, W doping and the 2H/1T structure were supposed to synergistically enhance the N_2_ adsorption. Binary and ternary metal sulfide-based composites were shown to facilitate photocatalytic NH_3_ synthesis via intimate heterointerfaces to diminish charge recombination (Dong Liu et al., 2021). Reported systems in this regard include MoS_2_/C-ZnO (Xing et al., 2018), C_3_N_4_/MoS_2_/Mn_3_O_4_ (Gui Li et al., 2021), WS_2_@TiO_2_ (Shi et al., 2020b), and MoS_2_/MgIn_2_S_4_ (Swain et al., 2020).

Sparked by the nitrogenase MoFe-based protein, the reduction of N_2_ on Fe single-atom-modified MoS_2_ nanosheet photocatalyst was first theoretically predicted by Azofra *et al.* (Azofra et al., 2017). Following this, various photocatalyst systems entailing Fe supported on MoS_2_ have emerged. As an example, Zheng *et al.* (Zheng et al., 2021) developed Fe-decorated 2D MoS_2_ photocatalysts [Fe-S_2_-Mo] mimicking FeMoco in nature for NH_3_ synthesis. A solar-to-NH_3_ energy-conversion efficiency of 0.24% at 270°C was achieved, representing the highest efficiency among all reported photocatalytic systems thus far. The HAADF-STEM images and simulation results jointly supported that Fe atoms were favorably situated on the atop sites of Mo rather than substituted Mo sites to form inorganic Fe-S_2_-Mo motifs analogous [Fe–S_2_–Mo] unit in FeMoco. Theoretical calculations suggested that the HOMO and LUMO orbitals were concentrated on the edge of single-layered MoS_2_ (sMoS_2_) with relatively low electron delocalization, indicating the active edge sites of sMoS_2_. Fe doping distinctly improved their LUMO orbital delocalization degree. Further *in situ* attenuated total reflection FTIR and the energy plots revealed that the NRR on Fe_1_ over [Fe–S_2_–Mo] followed an alternating pathway, showing similarity for both non-biological and biological processes regarding NRR mechanism. Excited electrons could be transferred from the VB to the CB of sMoS_2_ via the conductive Fe–S_2_–Mo motifs and reside on the Fe atom during the photoexcitation process to enter into the anti-bonding orbital of an adsorbed N_2_ molecule, which thus facilitated the hydrogenation reaction of N_2_ for ammonia production. Similarly, a biomimetic “MoFe cofactor” (the Fe^3+^/Fe^2+^ and Mo^6+^/Mo^4+^ redox couples) was introduced in MoTe_2_ nanosheets to facilitate the transport and separation of photo-generated charge carriers by one-electron and two-electron redox reactions with 15 times longer photocarrier lifetime after Fe doping and about 11 times higher NH_3_ production rate of Fe-doped MoTe_2_ than that of pure MoTe_2_ (Hongda Li et al., 2020). An enzymatic-analogous N_2_-fixation mechanism was also demonstrated on a bimetallic Ru–Co center at Ru/CoS_*x*_ interface on *g*-C_3_N_4_ sheets by Ru deposition near CoS_*x*_ induced by S vacancies (Figure 11C). The side-on bridging of N_2_ on under-coordinated Ru–Co center at Ru/CoS_*x*_ interface led to high polarization and strong activation of N_2_, resulting in an AQE of 1.28% at 400 nm and a STA efficiency of 0.042% for NH_3_ production in pure water.

### 3.4 Biomimetic photocatalysts

**FIGURE 12:**
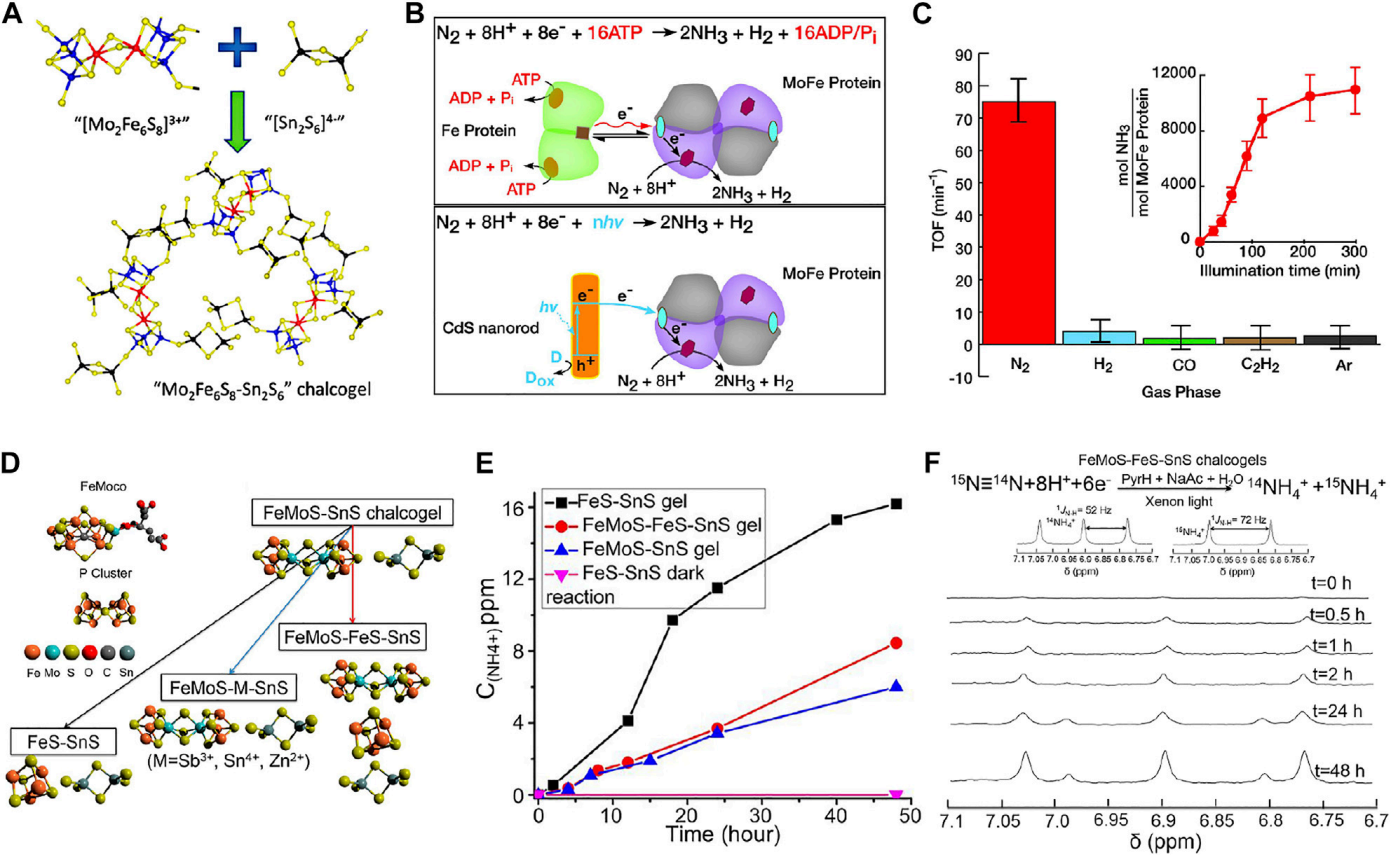
**(A)** Schematic of synthesis of Mo_2_Fe_6_S_8_–Sn_2_S_6_ biomimetic chalcogel (FeMoS-chalcogel) (Mo in blue; Fe in red; S in yellow; Sn in black). Reproduced from Banerjee et al. (2015) with permission from American Chemical Society. **(B)** Reaction scheme for photocatalytic NRR to NH_3_ by nitrogenase (top) and the CdS:MoFe protein biohybrids (below). **(C)** Photocatalytic NRR to NH_3_ on CdS:MoFe protein biohybrids under a 100% N_2_ atmosphere, 10% of either H_2_, CO, or C_2_H_2_ in a bulk phase of 90% N_2_. Reproduced from Brown et al. (2016) with permission from American Association for the Advancement of Science. **(D)** Model structures of the FeMo cofactor and P cluster, and the synthetic routes towards the assembly of different chalcogels. **(E)** Comparisons of photocatalytic NRR performance for different biomimetic chalcogels. **(F)** Isotope labeling experiment results detected by NMR. Reproduced from Liu et al. (2016) with permission from National Academy of Sciences of the United States of America.

Biological nitrogen fixation has advantages of low energy consumption and high NH_3_ yield, while multiple adenosine 5′-triphosphate (ATP) hydrolysis electron transfer steps are required per reduced N_2_ molecule, resulting in modest overall reaction kinetics and slow NH_3_ synthesis rate. Under such circumstance, exploration of biomimetic systems of molecular analogs to simulate and optimize this process has gained a tremendous interest in scientific community (Meng et al., 2021). Taking advantage of iron molybdenum sulfide chalcogels, Kanatzidis *et al.* (Banerjee et al., 2015) proposed a nitrogen-fixing biomimetic system by replacing MoFe-based proteins, which is the active site of nitrogenase, with Fe_2_Mo_6_S_8_ chalcogel interconnected through [Sn_2_S_6_]^4−^ ligands (Figure 12A). Featuring strong visible-light-absorbing, high spatial density of active sites, and multielectron transformations, the chalcogel-based amorphous framework could effectively convert N_2_ to NH_3_ in aqueous media under light illumination. Although its turnover number (TON) was not appealing, this study proved that cluster compounds analogous to nitrogenase can confer cogent catalysis and better stability than nitrogenase. Brown *et al.* (Brown et al., 2016) employed CdS to photosensitize MoFe proteins by harvesting light energy to replace ATP hydrolysis to drive the enzymatic N_2_ fixation, with peak NH_3_ production rates of 315 ± 55 nmol NH_3_ mg_(MoFe protein)_^−1^ min^−1^ at a TOF of 75 min^−1^ (Figures 12B,C). N_2_ reduction persisted for up to 5 h under constant illumination with a TON of 1.1 × 10^4^ mol NH_3_ mol_(MoFe protein)_^−1^. This study indicates that bio-nanocomposites can function as photocatalysts for solar-powered generation of NH_3_ with TOF comparable to nitrogenase. Inspired by the above works, Liu *et al.* (Liu et al., 2016) designed a redox-active FeMoS–FeS–SnS chalcogel system consisting of Fe_2_Mo_6_S_8_(SPh)_3_ and Fe_3_S_4_ biomimetic clusters linked by Sn_2_S_6_ to reduce N_2_ to NH_3_ (Figure 12D). All FeMoS–M–SnS chalcogels constructed by replacing Fe_4_S_4_ clusters with redox-inert ions Sb^3+^, Sn^4+^, Zn^2+^, exhibited effective NRR performance. FeMoS with FeS clusters was observed to strengthen NH_3_ production over FeMoS alone and Fe_4_S_4_-only chalcogel (FeS–SnS) and provided higher efficiency than that of [Mo_2_Fe_6_S_8_(SPh)_3_]-containing chalcogels. Therefore, Fe was believed to be more conducive than Mo for N_2_ binding (Figure 12E). The active sites in the Fe-containing sulfide clusters were considered to differ (i.e., based mainly on Fe) from that in the nitrogenase enzymes (based on both Mo and Fe). Quantitative isotope labeling and *in situ* DRIFTS corroborated the origin of detected NH_3_ from N_2_ (Figure 12F).

### 3.5 Metal-organic frameworks (MOFs)

**FIGURE 13:**
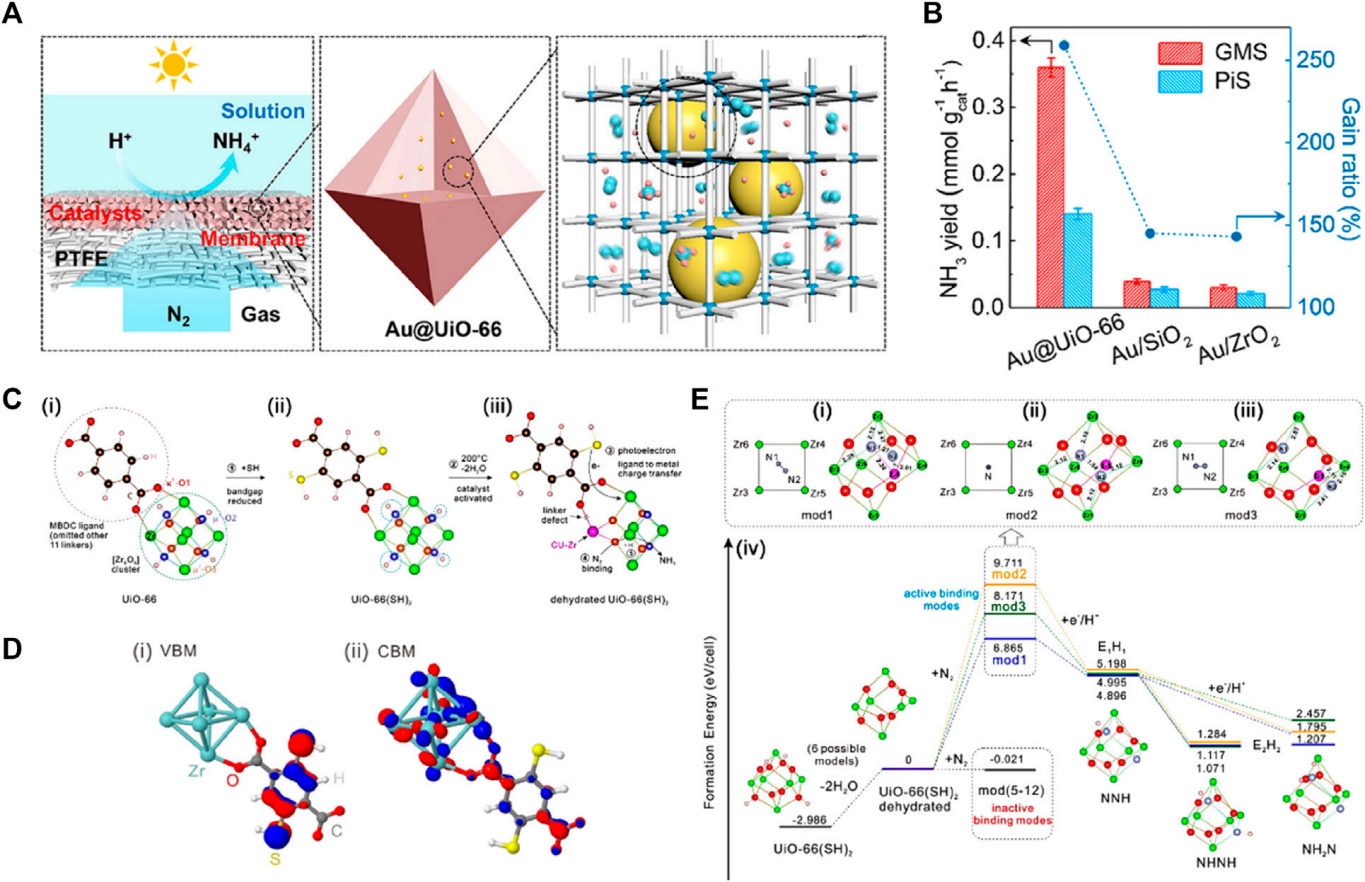
**(A)** Schematic illustration for direct NRR on Au encapsulated in UiO-66 matrix. **(B)** NH_3_ yield on powder (in the PiS system) and membrane (in the GMS system) catalysts consisting of Au@UiO-66, Au/SiO_2_, and Au/ZrO_2_. Reproduced from Chen et al. (2021b) with permission from American Chemical Society. **(C)** Schematic evolution of the partial structure of UiO66, UiO-66(SH)_2_ and the dehydrated UiO-66(SH)_2_. **(D)** The calculated band orbitals for VBM and CBM of UiO-66(SH)_2_ at Γ point. **(E)** The three active modes (mod 1–3) with each centerpiece configuration of the [Zr_6_O_6_] cluster encaging N_2_ molecule and their corresponding formation energy diagrams. Reproduced from Guo et al. (2022) with permission from the John Wiley and Sons.

MOFs are micro-mesoporous hybrid materials composed of metal ion nodes connected with organic linkers together or clusters and organic frameworks. Similar excitation characteristics of electron-hole pairs endow MOFs and their derivatives with intriguing semiconductor-like properties in various photochemical reactions (Hu et al., 2022). High microporosity and diverse functionalities enable the introduction of defined and highly exposed metal nodes onto the larger surfaces to promote or catalyze targeted reactions (Hao et al., 2021; Lin Wang et al., 2022). Especially, these exposed coordinatively unsaturated metal sites not only contribute to higher catalytic activities, but also behave as Lewis acid sites to withdraw *p* electrons from N_2_ molecules and weaken the N≡N triple-bonds, accounting for photochemical N_2_ reduction (Fu et al., 2022). Chen *et al.* (2021b) exploited gas-permeable MOF substrates (i.e., UiO-66) to not only serve as a stable matrix to confine the surface-clean gold nanoparticles (AuNPs) with high dispersity, but also ensure the accessibility of these AuNPs to both N_2_ molecules and (hydrated) protons (Figure 13A), enabling direct plasmonic NRR with high efficiency. The porosity of the MOF matrix facilitated mass transport of reactants and products, promoting the total reaction rate (Chen et al., 2022; Guanhua Zhang et al., 2022). Accordingly, the NH_3_ evolution rates on porous Au@MOFs particles were superior to those of nonporous particles at the specially designed gas–membrane–solution (GMS) reaction interface, and the GMS system was better than the powder-in-solution (PiS) system (Figure 13B). In another work, Guo *et al.* (Guo et al., 2022) investigated the [Zr_6_O_6_] cluster effect and the leading role of photoelectrons over the protonation of nitrogen by using an N-free dehydrated Zr-based MOF, UiO-66(SH)_2_ (Figure 13C). The UV/Vis diffuse reflectance spectrum combined with theory studies suggested that the introduction of thiol groups (–SH) caused an absorption edge of UiO-66(SH)_2_ deep into the visible region. The hopping process of the photoelectron from VB to the unoccupied Zr-4*d*ays is dominated by the ligand-to-metal charge transfer (LMCT) (Figure 13D). The dehydration opened a “gate” for the entry of N_2_ molecules into the [Zr_6_O_6_] cluster, of which three active cage modes strongly bound with N_2_ molecules and drive the cleavage of N≡N bond by the photoelectrons (Figure 13E). However, most MOF-related photocatalysts focused either on the coordination environment around the metal nodes, or the role of photosensitive ligands or single transition metals (Hu et al., 2022). Gao and co-authors presented photo-excited cluster defects and linker defects to revamp the photocatalytic NRR performance of UiO-66 (Gao et al., 2021). They shows the performance under alternate UV-Vis and visible light irradiation and after subsequent post-synthetic ligand exchange (PSE) process. Compared to UV-Vis light, the performance of the second test under visible light did not improve. After the PSE process that repaired linker defects instead of cluster defects, activity was restored under UV-Vis light. This demonstrated that photo-induced defects can only be created by UV light, where linker defects played a critical role in improving the performance due to the Zr nodes with unsaturated coordination induced by linker defects being more conducive to photo-driven NH_3_ synthesis.

Designing and constructing photocatalysts with bimetallic active centers is an effective way for nitrogen photofixation (Zhen Zhao et al., 2022). An *et al.* (An et al., 2021) designed a series of modularized UiO-66-based MOFs, U(Zr-Hf)-X, with bimetallic Zr-Hf nodes and functionalized ligands, and demonstrated a tandem ligand-to-metal-to-metal electron transfer (LMMET) pathway. By independently manipulating the Zr/Hf molar ratio and substituent group of TPA, the optimal U (0.5Hf)-2SH (metal nodes: 0.5Zr: 0.5Hf; linkers: TPA-2SH) yielded an NH_3_ production rate up to 116.1 μmol h^−1^ g^−1^ under visible light. This was attributed to the broadening of the absorption spectrum to visible light by –2SH modification according to combined experiment-theory results. Although these results suggested that the synergistic effect of bimetallic Zr-Hf nodes was favorable for NRR performance, it is uncertain whether the TPA-2SH ligand contributed to charger transfer mechanism. The proposed NRR pathway of U (0.5Hf)-2SH was explored. The one-step two-photon excitation path strides across the uphill process of the electron transfer from TPA-2SH ligands to metal nodes in UiO-66. The Hf species served as an electron buffer tank to optimize the electron transfer, while the Zr species acted as the catalytic active site due to the difference in electrode potentials between Zr–O and Hf–O clusters. Meanwhile, Zr-dominated π-backbonding mechanism weakened the N≡N bond in absorbed N_2_ molecules via the electron transfer synergy to generate NH_3_. Nonetheless, research on photocatalytic NRR using MOFs is relatively rare, the existing MOF-based photocatalysts have still an exemplary significance regarding constructing other visible-light-driven MOF-based NRR photocatalysts.

## 4 Conclusions and future remarks

The photocatalytic NRR is an appealing alternative to the current industrial thermocatalytic Haber-Bosch process for NH_3_ production. It only requires solar energy and water, rendering the process environmentally friendly and highly sustainable. With a general awareness of emphasizing on green chemistry and sustainable development, this field is bound to have a significant and far-reaching impact on how humans understand and engage in the nitrogen cycle. Despite recent progresses that have been achieved in the field of photocatalytic NH_3_ synthesis, it is still in its infancy. A comparable conversion efficiency to the century-old Haber-Bosch process remains unsolved, especially on how to accentuate N_2_ activation and accelerate the kinetics of electron transfer to N_2_. Continuous efforts in the design and development of nontoxic, efficient, stable and low-cost catalysts minimizing the consumption of noble metals have obtained some positive advancements. Tungsten and related metal-based materials have shown enormous accolades as photocatalysts for NRR. However, there still remain many challenges to be addressed as follows:

1) The intrinsic mechanism and kinetic control of nitrogen fixation are still ambiguous and complex, warranting further investigations. Because of the complex multi-electron reactions involved and the presence of various intermediate species, combination of multiple accurate analytical methods and advanced characterization techniques are required. It is essential to develop *in-situ* techniques to study the adsorption structure of N_2_ and key intermediates on the surface of catalytic materials, providing useful information on the catalytic active center and its dynamic evolution during the reaction process.
2) Various environmental factors (such as cation effect, electric field effect, pH, hydrophobicity, and actual active site, *etc.*) (Zhou et al., 2022) can profoundly affect the photocatalytic NRR process. It is thus necessary to explore and clarify the synergistic effect among the semiconductor photocatalysts and mixed solvent systems to further reveal the multi-effects of collaborative catalytic mechanism.
3) Equally importantly, the cooperative mechanisms among heterostructure, element doping and interaction between components and role of each individual strategy in N_2_ photocatalysis remain to be elucidated. It is necessary to comprehensively use various design and control strategies of catalysts to combine element doping, defect construction, and structural design to increase active sites, suppress the recombination of photogenerated carriers, and enhance the adsorption and activation of nitrogen molecules in the NRR process.
4) Efficient utilization of solar energy is the focus of interest in photocatalysis. However, the utilization of light in the NIR and the far-infrared region above 1,000 nm for NRR photocatalysis is poor. To facilitate the practical utilization of NRR photocatalysis, the design of suitable photocatalysts for wide-range light harvest from ultraviolet to near-infrared regions is important. This may be realized by integration with NIR and even far-infrared responsive materials such as dye molecules and black phosphorus (Zhang et al., 2017), narrow band gap NIR harvesters as well as materials having surface plasmon resonance effect.
5) The extensive use of sacrificial agents leads to additional costs, which deteriorate the overall affordability of the environment. Therefore, some cheap sacrificial agents at this stage, such as starch, biomass, plastics, wastewater, *etc.*, could also be developed as sacrificial agents to further reduce the cost of large-scale application of N_2_ photocatalysis.
6) The attained SCE of N_2_ photoreduction is still less than 1%, far below the minimum standard of 10% needed to realize industrialization, and the stability during the reaction process is however still much below the requirements for long-term practical applications.
7) The photostability of semiconductor catalysts is another adverse issue that deserves much attention. The surfaces of many *n*-type and *p*-type semiconductors (such as sulphides) are susceptible to decomposition by photogenerated holes or electrons, decreasing the lifetimes of the photocatalysts. To address this issue, strategies such as coating with a second phase, surface passivation and functionalization and incorporation with cocatalysts can be employed (Sun et al., 2018).

To date, major endeavors in materials engineering toward N_2_ photocatalysis have been made on traditional semiconductors. Some emerging photocatalytic materials such as MOFs and covalent organic frameworks (COFs) have not been fully explored for photocatalytic N_2_ reduction. Meanwhile, the design of cation defects and further combined defects and investigation of their roles in MOFs in N_2_ reduction are rarely conducted, which deserves further studies. To synergistically promote N_2_ photocatalysis, integration of multiple design strategies (e.g., defect engineering and other modification strategies such as creation of Z-scheme heterostructures to separate ammonia production and water oxidation sites in space) is preferred. Additionally, combination of material engineering and external fields (e.g., microwaves, mechanical stress, temperature gradient, electric field, magnetic field, and coupled fields) is another promising strategy to further boost photocatalytic N_2_ reduction reactions.

Current research focuses mainly on the microstructure design of materials and the improvement of photocatalytic performance, while less attention was paid on the development of photocatalytic devices suitable for future applications and reliable cost accounting. The bridge between laboratory research and practical application development is crucial. To better understand the conditions required to reach practical industrial applications, we should compare photocatalytic NRR with current industrial Haber-Bosch reactions from various perspectives, such as reaction temperature and pressure, ammonia/nitrate output, pollution, etc. In an actual photoreactor, N_2_ would be separated from the air using standard cryogenic separation or membrane processes and would need to be recovered and recycled for cost reasons. Current Photocatalytic NRR is more carried out in aqueous solutions, which means that liquid-phase products can be directly obtained to produce liquid fertilizers that can be directly used and sold, but the subsequent separation of catalysts will further increase the energy cost of the entire process. Even if the final product is anhydrous ammonia for fuel feedstock, the purification of low concentrations of NH_3_ from the N_2_ stream adds to the energy-consuming step. Therefore, how to achieve higher product concentrations in flowing gas setups is an important target of this particular approach. From another point of view, this idea guides us to no longer pay attention to the NH_3_ yield as an indicator, but to focus on the integration of upstream synthesis and downstream applications to reduce energy consumption and pollution in the entire production process, to achieve the final goal of “carbon neutrality” faster.
