# Supplementary material for: Recent progress of photocatalysts based on tungsten and related metals for nitrogen reduction to ammonia
Source: Front Chem. 2022 Aug 22;10:978078. doi: 10.3389/fchem.2022.978078 (PMC9441816; doi:10.3389/fchem.2022.978078)
Supplement: Supplementary file 1 [file Table1.DOCX]

**Recent progress of** **photocatalysts based on** **tungsten and related metals for nitrogen reduction to ammonia**

Xiangchao Hui,^a^ Lijun Wang,^a^ Zhibo Yao,^a^ Leiduan Hao,^a,^ * Zhenyu Sun^a,*^

^a^ State Key Laboratory of Organic-Inorganic Composites, College of Chemical Engineering, Beijing University of Chemical Technology, Beijing 100029, People’s Republic of China

*Correspondence: sunzy@mail.buct.edu.cn (Z.Y. Sun); haold@@mail.buct.edu.cn (L.D. Hao)

**Table 1.** Summary of Tungsten and related photocatalysts reported for photocatalytic nitrogen reduction.

| **Photocatalyst** | **Light source** | **^14^N_2_ purity**  **(%)** | **Reaction medium** | **NH_3_ yield; AQE/AQY (%)** | **Testing method** | **^15^N_2_ isotope^a^** | **N_2_ purification** | **Control experiment** | **Stability** | **Ref. (year)** |
| --- | --- | --- | --- | --- | --- | --- | --- | --- | --- | --- |
| OV–WO_3_-600 | *λ* ≥ 400 nm; (Xe, 200 mW·cm^−2^) | 99.999 | Water; 25 °C | 132 μmol g^−1^ h^−1^; AQY: 0.13 (420 nm) | Nessler’s reagent and IC | Yes | No | Dark, Ar | 10 cycles; 6 h | (Hou et al., 2019) |
| 2D WO_3–_*_x_* | 420 nm (4 × 3 W LED) | 99.9 | Na_2_SO_3_/water (1 mM) | 82.41 μmol g_cat_^–1^ h^–1^; AQY: 0.86% (420 nm) | Nessler’s reagent | No | No | Dark, Ar | 5 cycles | (Yang et al., 2022) |
| NiP*_x_*-3DOM H*_x_*WO_3-_*_y_* | Full spectrum; (300 W Xe) | − | Methanol/water (4 vol%); 35 °C | 34 μmol g^–1^ h^–1^ | IC | No | No | Dark, water | 4 cycles | (Ren et al., 2021) |
| Mn-WO_3_ | 320 ~ 780 nm; (Xe, 250 mW·cm^−2^) | 99.999 | Water; 25 °C | 425 µmol g^–1^ h^–1^; AQE: 0.18% (450 nm) | Nessler’s reagent and IC | Yes | Yes | Dark, Ar; No water or catalyst | 5 cycles | (Zhang et al., 2021) |
| 3Mo-WO_3_ | 300 W Xe | 99.9999 | ethanol/water (1/4, v/v); RT | 370 µmol g^–1^ h^–1^; AQY: 0.84% (420 nm) | Nessler's reagent | No | No | No N_2_ or water | 1 h | (Mao et al., 2020) |
| Mo-W_18_O_49_ | Full spectrum; (300 W Xe) | 99.999 | Na_2_SO_3_/water (1 mM); RT | 195.5 μmol g ^−1^ h^−1^; AQE: 0.33% (400 nm) | Nessler's reagent and IC | Yes | No | Dark, No catalyst or N_2_, or water | 5 cycles; 4 h | (Zhang et al., 2018) |
| Au@Mo-W_18_O_49_ | Full spectrum; (300 W Xe) | ≥ 99.9 | Methanol/water (20vol%); RT | 399.24 μmol g^−1^ h^−1^; AQE: 0.611% (540 nm) | Nessler's reagent and IC | No | No | Dark, Ar | 5 cycles; 10 h | (Qiu et al., 2021) |
| W_18_O_49_ | Simulated solar light (AM 1.5G, 100 mW cm^−2^) | − | Water; 25 °C | 22.8 μmol L^−1^ g^−1^ h^−1^ | IC | No | No | Dark, Ar | 10 cycles; 120 h | (Ren et al., 2020) |
| 2% Co-W_18_O_49_ | 300 W Xe | − | Ethylene glycol/water (1 mL); RT | 2752 μg^−1^ g ^−1^ | Nessler's reagent | No | No | No | No | (Ge et al., 2019) |
| Mn-W_18_O_49_ | Full spectrum; (300 W Xe) | − | Na_2_SO_4_/water (1.0 mM); RT | 97.9 μmol g^−1^ h ^−1^; AQE: 0.14% (350 nm) | Indophenol blue | No | No | Ar | 5 cycles; 20 h | (Ying et al., 2019) |
| W_18_O_49_/g-C_3_N_4_ | Simulated solar light (300 W Xe and 200 W IR) | − | Ethanol/water (0.789 g L^−1^) | 2.6 mg L^−1^ h^−1^ g^−1^ | Nessler's reagent | No | No | Dark, No catalyst or N_2_ | 40 h | (Liang et al., 2017) |
| Ag-g-C_3_N_4_/W_18_O_49_ | Simulated solar light; (300 W Xe and 200 W IR) | − | Ethanol/water (0.789 g L^−1^) | 3.2 mg L^−1^ h^−1^ g ^−1^ | Nessler's reagent | No | No | Dark, Ar; No catalyst or N_2_ | 40 h | (Liang et al., 2017) |
| Sb_2_O_3_/W_18_O_49_ | Vis; (300 W Xe) | 99.999 | Methanol/water (20 vol%); RT, 6 bar | 731 μg h^−1^ g^−1^; AQY: 0.063% (520 nm) | IC, ISE, and IC | No | Yes | Dark, Ar; No catalyst or N_2_ | 5 cycles; 3 h | (Hui et al., 2022) |
| W_18_O_49_/attapulgite | Simulated sunlight; (300 W Xe and NIR light) | − | Na_2_SO_3_/water (1 mM); RT | 138.76 μmol g^–1^ h^–1^ | Nessler's reagent | No | No | No | 2 h | (Liu et al., 2019) |
| WS/TiO_2_ film | Simulated sunlight (AM1.5) | − | Na_2_SO_3_/water (0.01 M); RT | 1.39 mmol g^–1^ h^–1^ | Indophenol blue | No | No | No | 4 cycles; 36 h | (Shi et al., 2020) |
| OV-Bi_2_WO_6_ | λ > 420 nm, (500 W Xe) | − | Water; RT | 471.13 μmol g^–1^ L^–1^ h^–1^ | IC | No | No | Dark, Ar | 4 cycles; 4 h | (Feng et al., 2022) |
| Hierarchical Bi_2_WO_6_ | 300 W Xe | − | Methanol/water (1 vol%); RT | 948 μmol g^−1^ h^−1^ | Nessler's reagent | No | No | Ar | No | (Bao et al., 2021) |
| B_i3.84_W_0.16_O_6.24_/g-C_3_N_4_ | Vis; (100 W Tungsten, 346 W/cm^–2^) | 99.9 | Ethanol/water (10 vol%); RT | 535.85 μmol L^–1^ h^–1^ | Nessler's reagent | No | No | Ar; No catalyst or N_2_, or water | 4 cycles | (Shende et al., 2019) |
| OV-Bi_2_WO_6_ | Simulated sunlight; (Xe, 400 mW∙cm^−2^) | 99.999 | Water; RT | 53.2 μmol g^–1^ h^–1^; AQY: 0.18% (350 nm) | Nessler's reagent and IC | Yes | No | Dark, air, Ar | 5 cycles; 10 h | (Wang et al., 2021) |
| GQDs/Bi_2_WO_6_ | Full-spectrum; (300 W Xe) | − | Ethanol/water (7.89 mg/L) | 48.21 μmol g^–1^ h^–1^ | Nessler’s reagent | No | No | No | 4 cycles | (Fei et al., 2019) |
| Bi_2_WO_6_/c-PAN | *λ* ≥ 420 nm; (300 W Xe) | − | Water; 20 °C | 140 μmol h^–1^ g^–1^ | Indophenol blue | No | No | Ar, No water | No | (Zhang et al., 2018) |
| Bi/Bi_2_WO_6_ nano-disks | Full-spectrum; (300 W Xe) | − | Water; 20 °C | 86 μmol g^–1^ h^–1^ | Nessler’s reagent | No | No | Dark, Ar; No N_2_ | 4 cycles | (Zhou et al., 2019) |
| Carbon-tungstic acid hybrids | Xe, 500 mW cm^−2^ | Air | Water | 220 μmol g^–1^ h^–1^ | Nessler's reagent | No | No | No | 60 min | (Li et al., 2016) |
| gC_3_N_4_/Cs*_x_*WO_3_ | 365 **~** 940 nm; (LED 100 mW cm^−2^) | − | Methanol/water (10 vol%); RT | 331 μmol h^−1^ g^−1^; AQY: 3.40% (365 nm) | Nessler’s reagent and IC | No | No | Dark, Ar | 10 cycles; 50 h | (Shi et al., 2018) |
| Fe-SrWO_4_ | Full-spectrum; (300 W Xe) | − | Water | 150.70 µM g^–1^ h^–1^ | Indophenol blue | No | No | Dark + No catalyst | 4 cycles | (Li et al., 2020) |
| SiW_12_/k-C_3_N_4_ | Vis; (Xe, 100 mW cm^−2^) | − | Water | 353.2 µmol·h^−1^ g^−1^ | Nessler's reagent | No | No | No | 6 h | (Xiao et al., 2018) |
| SiW_12_@MIL-101(Cr) | 300 W Xe | Air | Water | 75.56 μmol h^−1^ g^−1^ | Nessler’s reagent and IC | Yes | No | Dark, Ar | 5 cyles | (Su et al., 2022) |
| WC-Co/NGC | Full-spectrum; (300 W Xe) | − | Na_2_SO_3_/water (1 mM); RT | 157 μmol g^–1^ h^–1^ | Indophenol blue | Yes | No | Dark, Ar | 5 cyles | (Wang et al., 2021) |
| plasmonic MoO_3−_*_x_* spheres | Full-spectrum; (300 W Xe) | − | Methanol/water (20 vol%); | 435.57 µmol g^–1^ h^–1^; AQE: 1.24% (808 nm) | Nessler's reagent | No | No | Dark, Ar | 4 cycles; 6 h | (Bai et al., 2022) |
| MoO_3−_*_x_* nanosheet | *λ* ≥ 420 nm; (300 W Xe) | − | Water; 25 °C | 328 μM g^–1^ h^–1^; AQE: 0.31 (808 nm) | Nessler’s reagent and IC | No | No | No | 4 cycles | (Wu et al., 2020) |
| MoO_3-x_ nanobelts | Full-spectrum; (300 W Xe) | − | Water; 25 °C | 1.1µmol·h^−1^ g^−1^; AQE:  0.013% (365 nm) | IC | No | No | Ar | 3 cycles; 12 h | (Li et al., 2019) |
| MoO_2_/BiOCl | Xe, 500 mW cm^−2^ | − | Water | 35µmol g^–1^·h^–1^ | Nessler’s reagent | No | No | Dark, Ar | 5 cycles | (Xiao et al., 2019) |
| H–Bi_2_MoO_6_ sphere | *λ* ≥ 420 nm; (300 W Xe) | Purified air | Water; RT | 1.3 mmol g^–1^ h^–1^; AQE: 0.73 (500 nm) | Nessler’s reagent | No | No | Ar | 13 cycles | (Hao et al., 2016) (2016) |
| Mo SAC@PCN | 300 W Xe | − | Ethanol/water; RT | 830 μmol g^–1^ h^–1^; AQE: 0.70% (400 nm) | Nessler’s reagent and IC | Yes | No | Dark, Ar | 3 cycles; 36 h | (Guo et al., 2019) |
| Fe/Mo bimetallene–coated Bi_2_Mo_0.3_W_0.7_O_6_ | *λ* > 420 nm; (300 W Xe) | 99.999 | Water | 218.93 μmol g^–1^ h^–1^ | Nessler’s reagent | Yes | No | Ar | 4 cycles; 8 h | (Li et al., 2022) |
| OV-Bi_2_MoO_6_ | *λ* ≥ 420 nm; (300 W Xe) | − | Methanol/water (20 vol%); RT | 800 μmol·g^–1^ h^–1^; AQE: 5.2% (435 nm) | Nessler’s reagent and indophenol blue | No | No | Dark, Ar | 5 cycles; 10 h | (Li et al., 2021) |
| OV-P-Bi_2_MoO_6_ | Full-spectrum; (300 W Xe) | − | Water; 20 °C | 73.6 μmol g^–1^ h^–1^ | Nessler’s reagent | No | No | Air, Dark | 4 cycles | (Liu et al., 2021) |
| OV-Br-Bi_2_MoO_6_ | *λ* ≥ 420 nm; (300 W Xe) | − | Water; 20 °C | 1.60 μmol h^−1^; AQY: 0.52% (420 nm) | Nessler’s reagent | Yes | No | No | 5 cycles; 400 min | (Wang et al., 2022) |
| C-doped Bi_2_MoO_6_@attapulgite | Full-spectrum; (300 W Xe, 300 mW cm^−2^) | − | Ethanol/water (5 vol%) | 83.09 μmol g^–1^ h^–1^ | Nessler’s reagent | No | No | No | 5 cycles; 15 h | (Liu et al., 2020) |
| Bi_2_MoO_6_/OV-BiOBr | Full-spectrum; (300 W Xe) | − | Water; 25 °C | 90.7 μmol g^–1^ h^–1^ | Nessler’s reagent | No | No | No | 6 cycles | (Xue et al., 2019) |
| Fe-Bi_2_MoO_6_ | *λ* > 400 nm; (300 W Xe) | − | Water | 106.5 μmol g^–1^ h^–1^ | Nessler's reagent, indophenol blue and IC | No | No | Air, Ar; No catalyst or N_2_ | 5 cycles | (Meng et al., 2019) |
| gC_3_N_4_ /Bi_2_MoO_6_ | λ > 420 nm; (500 W Xe, 100 mW cm^−2^) | Air | Ethanol/water (0.789 g/L) | 3271 µmol L^−1^ g^−1^ | Nessler's reagent | No | No | No water | 4 cycles; 12 h | (Vesali-Kermani et al., 2020) |
| SiW_9_Co_3_/PD /Bi_2_WO_6_ | Simulated sunlight; (300 W Xe) | 99.999 | Water; RT | 52.5 μmol g^–1^ h^–1^ | Nessler's reagent, indophenol blue, and IC | Yes | No | Dark, Ar; No catalyst | 5 cycles; 10 h | (Wang et al., 2020) |
| Bi_3_FeMo_2_O_12_ | 450 W Hg | − | Water | 160.32 μmol g^–1^ h^–1^ | Indophenol blue | No | No | Dark, Ar | No | (Liu et al., 2019) |
| Fe-SrMoO_4_ | Full spectrum; (300 W Xe) | − | Water; 15 °C | 93.1 μM g^–1^ h^–1^ | and nessler’s reagent | No | No | Dark, No catalyst | 3 cycles | (Luo et al., 2019) |
| ZnO/Sb_2_MoO_6_ | Vis; (500 W Xe) | − | Ethanol/water (0.1 vol%); 25 °C | 2800 μmol g^–1^ L^–1^ | Nessler’s reagent | No | No | Dark, Ar; No water or catalyst | 4 cycles | (Mousavi et al., 2022) |
| 2H/1T Mixed-Phase Mo_1–_*_x_*W*_x_*S_2_ | > 400 nm; (300 W Xe) | 99.999 | Na_2_SO_3_/water (1 mM); 25 °C | 111 μmol g^–1^ h^–1^; AQY: 0.09% (420 nm) | Nessler’s reagent | Yes | No | Ar | 6 cycles; 18 h | (Qin et al., 2021) |
| MoS_2_/MgIn_2_S_4_ | Vis; (250 W Hg) | − | Water; RT | 0.405 mg L^–1^ h^–1^ | Indophenol blue | No | No | Dark, Ar; No water or catalyst | 4 cycles; 8 h | (Swain et al., 2020) |
| MoS_2_/C-ZnO | Full-spectrum; (300 W Xe) | air | Ethanol/water (5 vol%) | 245.7 μmol·L^–1^ g^–1^ h^–1^ | Nessler’s reagent | No | No | Blank | 6 cycles; 24 h | (Xing et al., 2018) |
| Fe-doped MoTe_2_ nanosheets | λ > 420 nm; (300 W Xe, 400 mW cm^–2^) | 99.999 | Water; 25 °C | 129.08 μmol g^–1^ h^–1^ | Nessler’s reagent | Yes | No | Ar | 4 cycles; 8 h | (Li et al., 2020) |
| CdS:MoFe protein | 405 nm; (25 mW cm^–2^) | 100 | 500 mM HEPES | 315 µmol g^−1^ min^−1^; AQY: 3.3% | Biovision, fluorescence assay | No | No | Ar | 5 h | (Brown et al., 2016) |
| [Mo_2_Fe_6_S_8_ (SPh)_3_]^3+^-[Sn_2_S_6_]^4−^ | Full-spectrum; (150 W Xe, 100 mW cm^–2^) | − | 5 mM ascorbic acid, 50 mM pyridinium hydrochloride in 10 mL aqueous solution; 25 °C | 5.21 mg L^−1^ | Indophenol blue, IC | Yes | No | Dark, Ar; No catalyst | 48 h | (Banerjee et al., 2015) |
| FeMoS-FeS-SnS chalcogel | λ >190 nm (150 W Xe, 100 mW cm^–2^) | − | 5 mM ascorbic acid, 50 mM pyridinium hydrochloride in 10 mL aqueous solution; RT | 10.5 mg L^−1^ | 1 H NMR | Yes^b^ | No | Dark, No catalyst or N_2_ | 48 h | (Liu et al., 2016) |
| Fe-sMoS_2_ | 70 W UV cut-off tungsten lamp | − | H_2_O; 270 °C, 6 bar | 2105 μmol g^–1^ h^1^; AQE: 37.1% (432 nm) | 1 H NMR and ISE | Yes^b^ | Yes | No | 6 cycles; 30 h | (Zheng et al., 2021) |
| Diamond/Mo | 450 W Hg/Xe | 99.9997 | water with 0.01 M Na_2_SO_4_ | 3.4 μg h^–1^ | Indophenol blue | No | Yes | No | No | (Bandy et al., 2016) |
| Trion-induced MoS_2_ | λ > 420 nm; (500 W Xe) | − | Water; 25 °C | 325 μmol h^–1^ g^–1^ | Indophenol blue | Yes | No | Dark, No catalyst | 10 h | (Sun et al., 2017) |
| Mo_0.1_Ni_0.1_Cd_0.8_S | 400 ~ 800 nm; (250 W Na) | 98 | Water/ethanol (0.789 g L^−1^); 30 °C | 3.2 mg·L^–1^ g^–1^ h^–1^ | Nessler’s reagent | Yes | No | No | 20 h | (Hu et al., 2016) |
| 1T-MoS_2_/CdS | 780 nm > *λ* >420 nm; (Xe, AM 1.5 G) | 99.999 | Methanol/water (20 vol %); RT | 8220.83 μmol L^–1^ g^–1^·h^–1^; AQE: 4.424% | Nessler’s reagent | No | No | No | 3 cycles | (Sun et al., 2020) |
| C_3_N_4_/MoS_2_/Mn_3_O_4_ | *λ* ≥ 420 nm; (300 W Xe) | − | Na_2_SO_3_/Water; RT | 185 μmol g^–1^·h^–1^; AQE: 1.2 (420 nm) | Indophenol blue | Yes | No | Dark, Ar | No | (Li et al., 2021) |
| *g*-C_3_N_4_/ZnMoCdS | 400 ~ 800 nm; (250 W Na) | − | Water/ethanol; 30 °C | 3.5 mg L^–1^ h^–1^ g^–1^ | Nessler’s reagent | No | No | Dark, No catalyst or N_2_ | 20 h | (Zhang et al., 2016) |
| La SAC@MoO_3−_***_x_*** | 420 ~ 800 nm; (300 W Xe, 1.73 W cm^–2^) | 99.999 | Water; RT | 209.0 μmol h^–1^ g^–1^ | IC | Yes | No | Ar | 5 cycles; 400 min | (Liu et al., 2022) |
| RGO/CoO/Co_3_O_4_ | Simulated sunlight; (300 W Xe) | 99.999 | Water | 89.1 μmol g^–1^ h^–1^; AQE: 0.37% (400 nm) | Indophenol blue | Yes | No | No | 6 cycles; 48 h | (Lu et al., 2022) |
| CoO*_x_* QD@GDY | Full-spectrum; (300 W Xe) | − | 0.1 M Na_2_SO_4_ aqueous solution | 26502 μmol g^–1^ h^–1^; AQY: 8.73% (500 nm) | Indophenol blue | Yes | Yes | Dark, Ar; No water | 6 independent experiments; 10 h | (Liu et al., 2021) |
| Ru-CoS/*g*-C_3_N_4_ | *λ* ≥ 420 nm; (300 W Xe, 200 mW·cm^−2^) | 99.999 | Methanol/water (10 vol %); RT | 0.438 mmol·g^–1^ h^–1^; AQE: 1.28 (400 nm) | Indophenol blue | Yes | No | No N_2_ + Ar | 12 cycles | (Yuan et al., 2020) |
| Co doped g-C_3_N_4_ | 400 ~ 800 nm; (250 W Na) | − | Methanol/water (0.04 vol %); RT | 5.8 mg L^−1^ h^−1^ g^−1^ | Nessler’s reagent | Yes | No | Dark, No N_2_ or catalyst | 6 cycles; 20 h | (Wang et al., 2019) |
| OV-CuCo-MOFs | *λ ≥* 400 nm; (300 W Xe) | 99.9 | Water; 25°C | 287.76 ± 7.02 μmol g^–1^ h^–1^ | Nessler’s reagent and IC | Yes | Yes | Ar | 3 cycles; 3 h | (Zhao et al., 2022) |
| CoFe_2_O_4_/g-C_3_N_4_ | Full spectrum; (300 W Xe) | 99.999 | Methanol/water (5 vol%) | 313 mg L^–1^ g^–1^ | Nessler's reagent | No | No | No | 5 cycles; 15 h | (Zheng et al., 2020) |
| Ag/PW_10_V_2_/am-TiO_2−_*_x_* | Simulated sunlight; (300 W Xe) | − | Water; 20 °C | 212.45 μmol g^–1^ h^–1^; AQE: 1.42% (350 nm) | Nessler's reagent and IC | Yes | No | Dark, Ar; No catalyst | 8 cycles | (Feng et al., 2022) |
| ZIF-67@PMo_4_V_8_ | Full spectrum; (300 W Xe) | − | Ethanol/water (20 vol %); RT | 149.0 μmol L^–1^ h^–1^ | Nessler's reagent | No | No | Ar | 5 cycles | (Li et al., 2020) |
| LaCoO_3_:Er^3+^/ATP | *λ* ≥ 420 nm; (300 W Xe) | − | Ethanol/water (5 vol%); 30 °C | 71.51 μmol g^–1^ h^–1^ | Nessler's reagent | No | No | — | No | (Zhang et al., 2019) |
| SV-BiVO_4_/ZnIn_2_S_4_ | λ > 400 nm; (300 W Xe 3.82 W cm^−2^) | 99.999 | Water; RT | 80.6 μmol g^–1^ h^–1^ | Nessler reagent and IC | Yes | No | Dark, No water or catalyst | 3 cycles | (Zhang et al., 2022) |
| BiVO_4_ | 400 ~ 800 nm; (300 W Xe) | − | Water | 103.4 μmol g^–1^ h^–1^ | Nessler's reagent | Yes | No | Dark, Ar; No water | No | (Zhang et al., 2021) |
| MnCO_3_/C-decorated BiVO_4_ | 300 W Xe, 100 mW cm^–2^ | − | Li_2_SO_4_/water (0.1 M) | 2.426 mmol m^−2^ h^−1^ | Nessler's reagent | Yes | No | Dark, Ar | 8 h | (Sun et al., 2020) |
| Ni/V-LDHs | (300 W Xe, 93 cW∙cm^−2^) | − | Warer; 25 °C | 176 µmol g^–1^ h^–1^ | IC | No | No | Ar | 3cycles | (Liu et al., 2021) |
| r-GO@PMo_10_V_2_ | Full-spectrum; (300 W Xe) | − | Warer; RT | 130.3 μmol L^–1^ h^–1^ | Nessler's reagent | No | No | O_2_, Ar | 5 cycles; 5 h | (Li et al., 2019) |
| Ni_3_V_2_O_8_/g-C_3_N_4_ | λ > 420 nm; (500 W Xe, 100 mW·cm^−2^) | − | Ethanol/water (0.02 vol%); 25 °C | 1118.5 μM h^−1^ g_cat_^−1^ | Nessler's reagent | No | No | Dark, No water or catalyst | 5 cycles | (Vesali-Kermani et al., 2020) |
| 10 wt % V-doped TiO_2_ | 400 W Hg | − | RT | 46.67 μmol L^–1^ g^–1^·h^–1^ | Nessler's reagent | No | Yes | Dark, Ar | No | (Ileperuma et al., 1993) |
| 1 wt% Fe-Tantalum oxide | UV; (450 W) | − | Water | 1.5 μmol | Indophenol blue | No | No | He | No | (Yue et al., 2005) |
| Bi_2_S_3_/KTa_0.75_Nb_0.25_O_3_ | 300 ~ 1000 nm; (300 W Xe, 54 mW cm^–2^) | 99.999 | Methanol/water (5 vol%) | 639.6 μmol L^–1^ g^–1^ h^–1^; AQE: 0.008% (400 nm) | 1H NMR | Yes^b^ | No | No N_2_ | 6 cycles; 30 h | (Chen et al., 2021) |
| KNbO_3_@TMU-5 | UV (0.2 W0 | − | Ethanol/water | 39.9 μmol·L^–1^ g^–1^ h^–1^ | Indophenol blue | No | No | Dark | 5 cycles; 30 h | (Chamack et al., 2022) |
| Ag/KNbO_3_ | Full-spectrum; (300 W Xe) | − | Ethanol/water (5 vol%) | 385.0 μmol·L^–1^ g^–1^·h^–1^ | Nessler’s reagent | No | No | Blank | 6 cycles; 30 h | (Xing et al., 2019) |
| NiO/KNbO_3_ | Simulated sunlight; (300 W Xe) | − | Ethanol/water (10 vol%) | 470.6 μmol g^–1^ h^−1^ | Nessler's reagent | No | No | Ar, No catalyst | 5 cycles; 25 h | (Xing et al., 2020) |
| LiNbO_3_/Pal | Simulated sunlight; (300 W Xe, 200 mW cm^–2^) | − | Water; 30 °C | 52.57 μmol g^–1^ h^−1^ | Nessler's reagent | No | No | No water | 6 cycles; 12 h | (Li et al., 2020) |
| B-HNbO_3_ NS | λ > 400 nm; (300 W Xe) | − | Water; RT | 170 μmol g^−1^ h^−1^; AQE: 0.64% (400 nm) | Indophenol blue and IC | Yes | No | Dark, Ar | 6 cycles | (Zhang et al., 2021) |
| Nb_2_O_5_/C/Nb_2_C/g-C_3_N_4_ | λ > 420 nm; (Xe, 0.5 W cm^–2^) | 99.999 | Methanol/water (20 vol%); RT | 365 μmol g^–1^ h^–1^ | Nessler's reagent | No | No | Dark, Ar; No N_2_ | 5 cycles; 10 h | (Jiang et al., 2020) |
| Au-NPs/Nb-SrTiO_3_/Zr/ZrO*_x_* film | 550 ~ 800 nm, Xe | − | Ethanol/water (10 vol%) | 0.007 μmol h^–1^ cm^–2^; AQY: 1% (600 nm) | Indophenol blue | Yes | No | No | 25 h | (Oshikiri et al., 2016) |
| UiO-66(SH)_2_ (Zr) | λ > 400 nm; (300 W Xe) | 99.999 | Water; 20, 40, 60, 80 °C | 32.28 μmol g^–1^ h^–1^; AQE: 0.45% (420 nm) | IC | Yes | No | Dark, Ar | 6 cycles; 6 h | (Guo et al., 2022) |
| U(0.5Hf)-2SH | λ > 420 nm; (300 W Xe) | − | K_2_SO_3_/water (0.79 g L^−1^) | 116.1 μmol g^–1^ h^–1^; AQY: 0.55% (420 nm) | IC | Yes | No | Dark, Ar | 5 cycles; 10 h | (An et al., 2021) |
| UiO-66(Zr) | UV-vis; (300 W Xe) | Air | Water | 196 μmol g^–1^ h^–1^ | Nessler’s reagent and IC | No | No | Dark, Ar | 5 cycles | (Gao et al., 2021) |
| Au@UiO-66 | λ > 400 nm; (300 W Xe, 100 mW cm^–2^) | 99.9999 | 0.5 K_2_SO_4_ aqueous solution; 25 °C | 18.9 mmol g_Au_^–1^ h^–1^; AQE; 1.54% (520 nm) | Nessler’s reagent and indophenol blue | Yes | No | Dark; No N_2_ or water, or catalyst | 6 cycles; 12 h | (Chen et al., 2021) |
| ZrO_2_/g-C_3_N_4_ | Full spectrum; (300 W Xe, 100 mW cm^–2^) | − | Methanol/water (10 vol%); 25 °C | 1446 μmol L^–1^ h^–1^; AQE: 2.14% (400 nm) | IC | Yes | No | Ar; No light or catalyst | 4 cycles; 16 h | (Mou et al., 2019) |
| Ag/PW_12_/Zr-*m*TiO_2_ | Simulated sunlight; (300 W Xe) | − | Water; 20 °C | 324.2 µmol g_cat_^−1^ h^−1^; AQE: 2.0% (350 nm) | Nessler’s reagent | Yes | No | Dark, Ar; No catalyst | 5 cycles | (Feng et al., 2022) |

Note: ^a^ only qualitatively, not quantitatively, unless otherwise stated; ^b^ refer to quantitative measurement.

**Reference**

Hou, T. T., Xiao, Y., Cui, P. X., Huang, Y., Tan, X. P., Zheng, X. S., et al. (2019). Operando oxygen vacancies for enhanced activity and stability toward nitrogen photofixation. *Adv. Energy Mater.* 9(43). doi:10.1002/aenm.201902319

Yang, Z., Wang, J., Wang, J., Li, M., Cheng, Q., Wang, Z., et al. (2022). 2D WO_3–_*_x_* nanosheet with rich oxygen vacancies for efficient visible-light-driven photocatalytic nitrogen fixation. *Langmuir* 38(3), 1178–1187. doi:10.1021/acs.langmuir.1c02862

Ren, X., Xia, M., Chong, B., Yan, X., Wells, N., and Yang, G. (2021). Uniform NiP*_x_* nanospheres loaded onto defective H*_x_*WO*_3-y_* with three-dimensionally ordered macroporous structure for photocatalytic nitrogen reduction. *Appl. Catal. B* 297, 120468. doi:10.1016/j.apcatb.2021.120468

Zhang, Y., Hou, T., Xu, Q., Wang, Q., Bai, Y., Yang, S., et al. (2021). Dual-metal sites boosting polarization of nitrogen molecules for efficient nitrogen photofixation. *Advanced Science* 8(13), 2100302. doi:10.1002/advs.202100302

Mao, Y., Yang, X., Gong, W., Zhang, J., Pan, T., Sun, H., et al. (2020). A dopant replacement-driven molten salt method toward the synthesis of sub-5-nm-sized ultrathin nanowires. *Small* 16(23), 2001098. doi:10.1002/smll.202001098

Zhang, N., Jalil, A., Wu, D. X., Chen, S. M., Liu, Y. F., Gao, C., et al. (2018). Refining defect states in W_18_O_49_ by Mo doping: A strategy for tuning N_2_ activation towards solar-driven nitrogen fixation. *J. Am. Chem. Soc.* 140(30), 9434–9443. doi:10.1021/jacs.8b02076

Qiu, P., Huang, C., Dong, G., Chen, F., Zhao, F., Yu, Y., et al. (2021). Plasmonic gold nanocrystals simulated efficient photocatalytic nitrogen fixation over Mo doped W_18_O_49_ nanowires. *J. Mater. Chem. A* 9(25), 14459–14465. doi:10.1039/D1TA03339E

Ren, W., Mei, Z., Zheng, S., Li, S., Zhu, Y., Zheng, J., et al. (2020). Wavelength-dependent solar N_2_ fixation into ammonia and nitrate in pure water. *Research (Wash D C)* 2020, 3750314. doi:10.34133/2020/3750314

Ge, J., Xu, J., Liu, Y., Zhang, L., Wang, L., and Wu, D. (2019). Enhanced nitrogen photo fixation performance of transition metal-doped urchin-like W_18_O_49_ under visible-light irradiation. *Nano* 14(11). doi:10.1142/s1793292019501431

Ying, Z., Chen, S., Zhang, S., Peng, T., and Li, R. (2019). Efficiently enhanced N_2_ photofixation performance of sea-urchin-like W_18_O_49_ microspheres with Mn-doping. *Appl. Catal. B* 254, 351–359. doi:10.1016/j.apcatb.2019.05.005

Liang, H., Zou, H., and Hu, S. (2017). Preparation of the W_18_O_49_/g-C_3_N_4_ heterojunction catalyst with full-spectrum-driven photocatalytic N_2_ photofixation ability from the UV to near infrared region. *New J. Chem.* 41(17), 8920–8926. doi:10.1039/c7nj01848g

Liang, H., Li, J., and Tian, Y. (2017). Construction of full-spectrum-driven Ag–g-C_3_N_4_/W_18_O_49_ heterojunction catalyst with outstanding N_2_ photofixation ability. *RSC Adv.* 7(68), 42997–43004. doi:10.1039/C7RA08420J

Hui, X., Li, L., Xia, Q., Hong, S., Hao, L., Robertson, A. W., et al. (2022). Interface engineered Sb_2_O_3_/W_18_O_49_ heterostructure for enhanced visible-light-driven photocatalytic N_2_ reduction. *Chem. Eng. J.* 438, 135485. doi:10.1016/j.cej.2022.135485

Liu, Y., Li, X., Su, H., Chen, X., Zuo, S., Qian, J., et al. (2019). Plasmonic W_18_O_49_/attapulgite nanocomposite with enhanced photofixation of nitrogen under full-spectrum light. *Journal of Materials Science: Materials in Electronics* 30(22), 20019–20028. doi:10.1007/s10854-019-02370-1

Shi, L., Li, Z., Ju, L., Carrasco-Pena, A., Orlovskaya, N., Zhou, H., et al. (2020). Promoting nitrogen photofixation over a periodic WS_2_@TiO_2_ nanoporous film. *J. Mater. Chem. A* 8(3), 1059–1065. doi:10.1039/C9TA12743G

Feng, Y., zhao, Z., Wang, T., Li, J., Xu, M., Jiao, H., et al. (2022). Magnetic field-enhanced photocatalytic nitrogen fixation over defect-rich ferroelectric Bi_2_WO_6_. *Ceram. Int.* doi:10.1016/j.ceramint.2022.03.282

Bao, L., Yuan, Y.-j., Zhang, H., Zhang, X., and Xu, G. (2021). Understanding the hierarchical behavior of Bi_2_WO_6_ with enhanced photocatalytic nitrogen fixation activity. *Dalton Trans.* 50(21), 7427–7432. doi:10.1039/D1DT00762A

Shende, A. G., Tiwari, C. S., Bhoyar, T. H., Vidyasagar, D., and Umare, S. S. (2019). BWO nano-octahedron coupled with layered g-C_3_N_4_: An efficient visible light active photocatalyst for degradation of cationic/anionic dyes, and N_2_ reduction. *J. Mol. Liq.* 296, 111771. doi:10.1016/j.molliq.2019.111771

Wang, T., Feng, C., Liu, J., Wang, D., Hu, H., Hu, J., et al. (2021). Bi_2_WO_6_ hollow microspheres with high specific surface area and oxygen vacancies for efficient photocatalysis N_2_ fixation. *Chem. Eng. J.* 414, 128827. doi:10.1016/j.cej.2021.128827

Fei, T., Yu, L., Liu, Z., Song, Y., Xu, F., Mo, Z., et al. (2019). Graphene quantum dots modified flower like Bi_2_WO_6_ for enhanced photocatalytic nitrogen fixation. *J. Colloid Interface Sci.* 557, 498–505. doi:10.1016/j.jcis.2019.09.011

Zhang, C., Chen, G., Lv, C., Yao, Y., Xu, Y., Jin, X., et al. (2018). Enabling nitrogen fixation on Bi_2_WO_6_ photocatalyst by c-PAN surface decoration. *ACS Sustain. Chem. Eng.* 6(9), 11190–11195. doi:10.1021/acssuschemeng.8b02236

Zhou, S., Zhang, C., Liu, J., Liao, J., Kong, Y., Xu, Y., et al. (2019). Formation of an oriented Bi_2_WO_6_ photocatalyst induced by in situ Bi reduction and its use for efficient nitrogen fixation. *Catal. Sci. Technol.* 9(20), 5562–5566. doi:10.1039/C9CY00972H

Li, X., Wang, W., Jiang, D., Sun, S., Zhang, L., and Sun, X. (2016). Efficient solar-driven nitrogen fixation over carbon–tungstic-acid hybrids. *Chem. Eur. J.* 22(39), 13819–13822. doi:10.1002/chem.201603277

Shi, A., Li, H., Yin, S., Hou, Z., Rong, J., Zhang, J., et al. (2018). Photocatalytic NH_3_ versus H_2_ evolution over g-C_3_N_4_/Cs*_x_*WO_3_: O_2_ and methanol tipping the scale. *Appl. Catal. B* 235, 197–206. doi:10.1016/j.apcatb.2018.04.081

Li, Q., Bai, X., Luo, J., Li, C., Wang, Z., Wu, W., et al. (2020). Fe doped SrWO_4_ with tunable band structure for photocatalytic nitrogen fixation. *Nanotechnology* 31(37), 375402. doi:10.1088/1361-6528/ab9863

Xiao, C., Zhang, L., Wang, K., Wang, H., Zhou, Y., and Wang, W. (2018). A new approach to enhance photocatalytic nitrogen fixation performance via phosphate-bridge: A case study of SiW_12_/K-C_3_N_4_. *Appl. Catal. B* 239, 260–267. doi:10.1016/j.apcatb.2018.08.012

Su, S., Li, X., Zhang, X., Zhu, J., Liu, G., Tan, M., et al. (2022). Keggin-type SiW_12_ encapsulated in MIL-101(Cr) as efficient heterogeneous photocatalysts for nitrogen fixation reaction. *J. Colloid Interface Sci.* doi:10.1016/j.jcis.2022.04.006

Wang, L., Zhang, Q., Wei, T., Li, F., Sun, Z., and Xu, L. (2021). WC and cobalt nanoparticles embedded in nitrogen-doped carbon 3D nanocage derived from H_3_PW_12_O_40_@ZIF-67 for photocatalytic nitrogen fixation. *J. Mater. Chem. A* 9(5), 2912–2918. doi:10.1039/D0TA10303A

Bai, H., Lam, S. H., Yang, J., Cheng, X., Li, S., Jiang, R., et al. (2022). A Schottky-barrier-free plasmonic semiconductor photocatalyst for nitrogen fixation in a “one-stone-two-birds” manner. *Adv. Mater.* 34(2), 2104226. doi:10.1002/adma.202104226

Wu, H., Li, X., Cheng, Y., Xiao, Y., Li, R., Wu, Q., et al. (2020). Plasmon-driven N_2_ photofixation in pure water over MoO_3−_*_x_* nanosheets under visible to NIR excitation. *J. Mater. Chem. A* 8(5), 2827–2835. doi:10.1039/C9TA13038A

Li, Y., Chen, X., Zhang, M., Zhu, Y., Ren, W., Mei, Z., et al. (2019). Oxygen vacancy-rich MoO_3−x_ nanobelts for photocatalytic N_2_ reduction to NH_3_ in pure water. *Catal. Sci. Technol.* 9(3), 803–810. doi:10.1039/C8CY02357C

Xiao, C., Wang, H., Zhang, L., Sun, S., and Wang, W. (2019). Enhanced photocatalytic nitrogen fixation on MoO_2_/BiOCl composite. *ChemCatChem* 11(24), 6467–6472. doi:10.1002/cctc.201901635

Hao, Y., Dong, X., Zhai, S., Ma, H., Wang, X., and Zhang, X. (2016). Hydrogenated bismuth molybdate nanoframe for efficient sunlight‐driven nitrogen fixation from air. *Chem. Eur. J.* 22(52), 18722–18728. doi:10.1002/chem.201604510

Guo, X.-W., Chen, S.-M., Wang, H.-J., Zhang, Z.-M., Lin, H., Song, L., et al. (2019). Single-atom molybdenum immobilized on photoactive carbon nitride as efficient photocatalysts for ambient nitrogen fixation in pure water. *J. Mater. Chem. A* 7(34), 19831–19837. doi:10.1039/C9TA06653E

Li, H., Deng, H., Gu, S., Li, C., Tao, B., Chen, S., et al. (2022). Engineering of bionic Fe/Mo bimetallene for boosting the photocatalytic nitrogen reduction performance. *J. Colloid Interface Sci.* 607, 1625–1632. doi:10.1016/j.jcis.2021.09.078

Li, G., Yang, W., Gao, S., Shen, Q., Xue, J., Chen, K., et al. (2021). Creation of rich oxygen vacancies in bismuth molybdate nanosheets to boost the photocatalytic nitrogen fixation performance under visible light illumination. *Chem. Eng. J.* 404, 127115. doi:10.1016/j.cej.2020.127115

Liu, L., Liu, J., Sun, K., Wan, J., Fu, F., and Fan, J. (2021). Novel phosphorus-doped Bi_2_WO_6_ monolayer with oxygen vacancies for superior photocatalytic water detoxication and nitrogen fixation performance. *Chem. Eng. J.* 411, 128629. doi:10.1016/j.cej.2021.128629

Wang, G., Huo, T., Deng, Q., Yu, F., Xia, Y., Li, H., et al. (2022). Surface-layer bromine doping enhanced generation of surface oxygen vacancies in bismuth molybdate for efficient photocatalytic nitrogen fixation. *Appl. Catal. B* 310, 121319. doi:10.1016/j.apcatb.2022.121319

Liu, W., Yin, K., Yuan, K., Zuo, S., Yang, S., Yao, C., et al. (2020). In situ synthesis of Bi_2_MoO_6_@C@attapulgite photocatalyst for enhanced photocatalytic nitrogen fixation ability under simulated solar irradiation. *Colloid Surface A* 591, 124488. doi:10.1016/j.colsurfa.2020.124488

Xue, X., Chen, R., Yan, C., Hu, Y., Zhang, W., Yang, S., et al. (2019). Efficient photocatalytic nitrogen fixation under ambient conditions enabled by the heterojunctions of n-type Bi_2_MoO_6_ and oxygen-vacancy-rich p-type BiOBr. *Nanoscale* 11(21), 10439–10445. doi:10.1039/C9NR02279A

Meng, Q., Lv, C., Sun, J., Hong, W., Xing, W., Qiang, L., et al. (2019). High-efficiency Fe-mediated Bi_2_MoO_6_ nitrogen-fixing photocatalyst: Reduced surface work function and ameliorated surface reaction. *Appl. Catal. B* 256, 117781. doi:10.1016/j.apcatb.2019.117781

Vesali-Kermani, E., Habibi-Yangjeh, A., Diarmand-Khalilabad, H., and Ghosh, S. (2020). Nitrogen photofixation ability of g-C_3_N_4_ nanosheets/Bi_2_MoO_6_ heterojunction photocatalyst under visible-light illumination. *J. Colloid Interface Sci.* 563, 81–91. doi:10.1016/j.jcis.2019.12.057

Wang, T., Liu, J., Wu, P., Feng, C., Wang, D., Hu, H., et al. (2020). Direct utilization of air and water as feedstocks in the photo-driven nitrogen reduction reaction over a ternary Z-scheme SiW_9_Co_3_/PDA/BWO hetero-junction. *J. Mater. Chem. A* 8(32), 16590–16598. doi:10.1039/C9TA13902H

Liu, B., Yasin, A. S., Musho, T., Bright, J., Tang, H., Huang, L., et al. (2019). Visible-light bismuth iron molybdate photocatalyst for artificial nitrogen fixation. *J. Electrochem. Soc.* 166(5), H3091–H3096. doi:10.1149/2.0151905jes

Luo, J., Bai, X., Li, Q., Yu, X., Li, C., Wang, Z., et al. (2019). Band structure engineering of bioinspired Fe doped SrMoO_4_ for enhanced photocatalytic nitrogen reduction performance. *Nano Energy* 66, 104187. doi:10.1016/j.nanoen.2019.104187

Mousavi, M., Habibi, M. M., Zhang, G., Pourhakkak, P., moradian, S., and Ghasemi, J. B. (2022). In-situ construction of ZnO/Sb_2_MoO_6_ nano-heterostructure for efficient visible-light photocatalytic conversion of N_2_ to NH_3_. *Surf. Interfaces* 30, 101844. doi:10.1016/j.surfin.2022.101844

Qin, J., Zhao, W., Hu, X., Li, J., Ndokoye, P., and Liu, B. (2021). Exploring the N_2_ adsorption and activation mechanisms over the 2H/1T Mixed-phase ultrathin Mo_1–x_W_x_S_2_ nanosheets for boosting N_2_ photosynthesis. *ACS Appl. Mater. Interfaces* 13(6), 7127–7134. doi:10.1021/acsami.0c19282

Swain, G., Sultana, S., and Parida, K. (2020). Constructing a novel surfactant-free MoS_2_ nanosheet modified MgIn_2_S_4_ marigold microflower: An efficient visible-light driven 2D-2D p-n heterojunction photocatalyst toward HER and pH regulated NRR. *ACS Sustain. Chem. Eng.* 8(12), 4848–4862. doi:10.1021/acssuschemeng.9b07821

Xing, P., Chen, P., Chen, Z., Hu, X., Lin, H., Wu, Y., et al. (2018). Novel ternary MoS_2_/C-ZnO composite with efficient performance in photocatalytic NH_3_ synthesis under simulated sunlight. *ACS Sustain. Chem. Eng.* 6(11), 14866–14879. doi:10.1021/acssuschemeng.8b03388

Li, H., Gu, S., Sun, Z., Guo, F., Xie, Y., Tao, B., et al. (2020). The in-built bionic “MoFe cofactor” in Fe-doped two-dimensional MoTe_2_ nanosheets for boosting the photocatalytic nitrogen reduction performance. *J. Mater. Chem. A* 8(26), 13038–13048. doi:10.1039/d0ta04251j

Brown, K. A., Harris, D. F., Wilker, M. B., Rasmussen, A., Khadka, N., Hamby, H., et al. (2016). Light-driven dinitrogen reduction catalyzed by a CdS: Nitrogenase MoFe protein biohybrid. *Science* 352(6284), 448–450. doi:10.1126/science.aaf2091

Banerjee, A., Yuhas, B. D., Margulies, E. A., Zhang, Y., Shim, Y., Wasielewski, M. R., et al. (2015). Photochemical nitrogen conversion to ammonia in ambient conditions with FeMoS-chalcogels. *J. Am. Chem. Soc.* 137(5), 2030–2034. doi:10.1021/ja512491v

Liu, J., Kelley, M. S., Wu, W., Banerjee, A., Douvalis, A. P., Wu, J., et al. (2016). Nitrogenase-mimic iron-containing chalcogels for photochemical reduction of dinitrogen to ammonia. *Proc. Natl. Acad. Sci.* 113(20), 5530–5535. doi:doi:10.1073/pnas.1605512113

Zheng, J., Lu, L., Lebedev, K., Wu, S., Zhao, P., McPherson, I. J., et al. (2021). Fe on molecular-layer MoS_2_ as inorganic Fe-S_2_-Mo motifs for light-driven nitrogen fixation to ammonia at elevated temperatures. *Chem Catal.* 1(1), 162–182. doi:10.1016/j.checat.2021.03.002

Bandy, J. A., Zhu, D., and Hamers, R. J. (2016). Photocatalytic reduction of nitrogen to ammonia on diamond thin films grown on metallic substrates. *Diamond Relat. Mater.* 64, 34–41. doi:10.1016/j.diamond.2016.01.006

Sun, S., Li, X., Wang, W., Zhang, L., and Sun, X. (2017). Photocatalytic robust solar energy reduction of dinitrogen to ammonia on ultrathin MoS_2_. *Appl. Catal. B* 200, 323–329. doi:10.1016/j.apcatb.2016.07.025

Hu, S., Chen, X., Li, Q., Zhao, Y., and Mao, W. (2016). Effect of sulfur vacancies on the nitrogen photofixation performance of ternary metal sulfide photocatalysts. *Catal. Sci. Technol.* 6(15), 5884–5890. doi:10.1039/C6CY00622A

Sun, B., Liang, Z., Qian, Y., Xu, X., Han, Y., and Tian, J. (2020). Sulfur vacancy-rich O-doped 1T-MoS_2_ nanosheets for exceptional photocatalytic nitrogen fixation over CdS. *ACS Appl. Mater. Interfaces* 12(6), 7257–7269. doi:10.1021/acsami.9b20767

Li, H., Liu, Y., Liu, Y., Wang, L., Tang, R., Deng, P., et al. (2021). Efficient visible light driven ammonia synthesis on sandwich structured C_3_N_4_/MoS_2_/Mn_3_O_4_ catalyst. *Appl. Catal. B* 281, 119476. doi:10.1016/j.apcatb.2020.119476

Zhang, Q., Hu, S., Fan, Z., Liu, D., Zhao, Y., Ma, H., et al. (2016). Preparation of g-C_3_N_4_/ZnMoCdS hybrid heterojunction catalyst with outstanding nitrogen photofixation performance under visible light via hydrothermal post-treatment. *Dalton Trans.* 45(8), 3497–3505. doi:10.1039/C5DT04901F

Liu, X., Luo, Y., Ling, C., Shi, Y., Zhan, G., Li, H., et al. (2022). Rare earth La single atoms supported MoO_3_*_−x_* for efficient photocatalytic nitrogen fixation. *Appl. Catal. B* 301, 120766. doi:10.1016/j.apcatb.2021.120766

Lu, H., Zhao, Y.-M., Saji, S. E., Yin, X., Wibowo, A., Tang, C. S., et al. (2022). All room-temperature synthesis, N_2_ photofixation and reactivation over 2D cobalt oxides. *Appl. Catal. B* 304, 121001. doi:10.1016/j.apcatb.2021.121001

Liu, Y., Xue, Y., Hui, L., Yu, H., Fang, Y., He, F., et al. (2021). Porous graphdiyne loading CoO*_x_* quantum dots for fixation nitrogen reaction. *Nano Energy* 89, 106333. doi:10.1016/j.nanoen.2021.106333

Yuan, J., Yi, X., Tang, Y., Liu, M., and Liu, C. (2020). Efficient photocatalytic nitrogen fixation: Enhanced polarization, activation, and cleavage by asymmetrical electron donation to N≡N bond. *Adv. Funct. Mater.* 30(4), 1906983. doi:10.1002/adfm.201906983

Wang, K., Gu, G., Hu, S., Zhang, J., Sun, X., Wang, F., et al. (2019). Molten salt assistant synthesis of three-dimensional cobalt doped graphitic carbon nitride for photocatalytic N_2_ fixation: Experiment and DFT simulation analysis. *Chem. Eng. J.* 368, 896–904. doi:10.1016/j.cej.2019.03.037

Zhao, W., Qin, J., Teng, W., Mu, J., Chen, C., Ke, J., et al. (2022). Catalytic photo-redox of simulated air into ammonia over bimetallic MOFs nanosheets with oxygen vacancies. *Appl. Catal. B* 305, 121046. doi:10.1016/j.apcatb.2021.121046

Zheng, Q., Xu, Y., Wan, Y., Wu, J., Hu, X., and Yao, X. (2020). Synthesis of CoFe_2_O_4_-modified g-C_3_N_4_ with enhanced photocatalytic performance for nitrogen fixation. *J Nanopart. Res.* 22(9), 301. doi:10.1007/s11051-020-05028-w

Feng, C., Wu, P., Li, Q., Liu, J., Wang, D., Liu, B., et al. (2022). Amorphization and defect engineering in constructing ternary composite Ag/PW_10_V_2_/am-TiO_2−x_ for enhanced photocatalytic nitrogen fixation. *New J. Chem.* 46(4), 1731–1740. doi:10.1039/D1NJ05917C

Li, X.-H., He, P., Wang, T., Zhang, X.-W., Chen, W.-L., and Li, Y.-G. (2020). Keggin-type polyoxometalate-based ZIF-67 for enhanced photocatalytic nitrogen fixation. *ChemSusChem* 13(10), 2769–2778. doi:10.1002/cssc.202000328

Zhang, H., Li, X., Su, H., Chen, X., Zuo, S., Yan, X., et al. (2019). Sol–gel synthesis of upconversion perovskite/attapulgite heterostructures for photocatalytic fixation of nitrogen. *J. Sol-Gel Sci. Technol.* 92(1), 154–162. doi:10.1007/s10971-019-05071-7

Zhang, G., Yuan, X., Xie, B., Meng, Y., Ni, Z., and Xia, S. (2022). S vacancies act as a bridge to promote electron injection from Z-scheme heterojunction to nitrogen molecule for photocatalytic ammonia synthesis. *Chem. Eng. J.* 433, 133670. doi:10.1016/j.cej.2021.133670

Zhang, G., Meng, Y., Xie, B., Ni, Z., Lu, H., and Xia, S. (2021). Precise location and regulation of active sites for highly efficient photocatalytic synthesis of ammonia by facet-dependent BiVO_4_ single crystals. *Appl. Catal. B* 296, 120379. doi:10.1016/j.apcatb.2021.120379

Sun, D., Bai, H., Zhao, Y., Zhang, Q., Bai, Y., Liu, Y., et al. (2020). Amorphous MnCO_3_/C double layers decorated on BiVO_4_ photoelectrodes to boost nitrogen reduction. *ACS Appl. Mater. Interfaces* 12(47), 52763–52770. doi:10.1021/acsami.0c16337

Liu, X., Li, Y., Zhang, J., and Lu, J. (2021). Ultrathin Ni/V-layered double hydroxide nanosheets for efficient visible-light-driven photocatalytic nitrogen reduction to ammonia. *Nano Res.* 14(10), 3372–3378. doi:10.1007/s12274-021-3641-3

Li, X.-H., Chen, W.-L., Tan, H.-Q., Li, F.-R., Li, J.-P., Li, Y.-G., et al. (2019). Reduced state of the graphene oxide@polyoxometalate nanocatalyst achieving high-efficiency nitrogen fixation under light driving conditions. *ACS Appl. Mater. Interfaces* 11(41), 37927–37938. doi:10.1021/acsami.9b12328

Vesali-Kermani, E., Habibi-Yangjeh, A., and Ghosh, S. (2020). Efficiently enhanced nitrogen fixation performance of g-C_3_N_4_ nanosheets by decorating Ni_3_V_2_O_8_ nanoparticles under visible-light irradiation. *Ceram. Int.* 46(15), 24472–24482. doi:10.1016/j.ceramint.2020.06.232

Ileperuma, O. A., Thaminimulla, C. T. K., and Kiridena, W. C. B. (1993). Photoreduction of N_2_ to NH_3_ and H_2_O to H_2_ on metal doped TiO_2_ catalysts (M = Ce, V). *Sol. Energy Mater. Sol. Cells* 28(4), 335–343. doi:10.1016/0927-0248(93)90121-I

Yue, C., Trudeau, M. L., and Antonelli, D. (2005). Mesoporous tantalum oxide photocatalysts for Schrauzer-type conversion of dinitrogen to ammonia. *Can. J. Chem.* 83(4), 308–314. doi:10.1139/v05-018

Chen, L., Dai, X., Li, X., Wang, J., Chen, H., Hu, X., et al. (2021). A novel Bi_2_S_3_/KTa_0.75_Nb_0.25_O_3_ nanocomposite with high efficiency for photocatalytic and piezocatalytic N_2_ fixation. *J. Mater. Chem. A* 9(22), 13344–13354. doi:10.1039/D1TA02270A

Chamack, M., Ifires, M., Akbar Razavi, S. A., Morsali, A., Addad, A., Larimi, A., et al. (2022). Photocatalytic performance of perovskite and metal-organic framework hybrid material for the reduction of N_2_ to ammonia. *Inorg. Chem.* 61(3), 1735–1744. doi:10.1021/acs.inorgchem.1c03622

Xing, P., Wu, S., Chen, Y., Chen, P., Hu, X., Lin, H., et al. (2019). New application and excellent performance of Ag/KNbO_3_ nanocomposite in photocatalytic NH_3_ synthesis. *ACS Sustain. Chem. Eng.* 7(14), 12408–12418. doi:10.1021/acssuschemeng.9b01938

Xing, P., Zhang, W., Chen, L., Dai, X., Zhang, J., Zhao, L., et al. (2020). Preparation of a NiO/KNbO_3_ nanocomposite via a photodeposition method and its superior performance in photocatalytic N_2_ fixation. *Sustain. Energy Fuels* 4(3), 1112–1117. doi:10.1039/C9SE01003C

Li, X., He, C., Dai, D., Zuo, S., Yan, X., Yao, C., et al. (2020). Nano-mineral induced nonlinear optical LiNbO_3_ with abundant oxygen vacancies for photocatalytic nitrogen fixation: Boosting effect of polarization. *Appl. Nanosci.* 10(9), 3477–3490. doi:10.1007/s13204-020-01443-6

Zhang, Y., Ran, L., Zhang, Y., Zhai, P., Wu, Y., Gao, J., et al. (2021). Two-dimensional defective boron-doped niobic acid nanosheets for robust nitrogen photofixation. *ACS Nano*. doi:10.1021/acsnano.1c06017

Jiang, H., Zang, C., Zhang, Y., Wang, W., Yang, C., Sun, B., et al. (2020). 2D MXene-derived Nb_2_O_5_/C/Nb_2_C/g-C_3_N_4_ heterojunctions for efficient nitrogen photofixation. *Catal. Sci. Technol.* 10(17), 5964–5972. doi:10.1039/D0CY00656D

Oshikiri, T., Ueno, K., and Misawa, H. (2016). Selective dinitrogen conversion to ammonia using water and visible light through plasmon‐induced charge separation. *Angew. Chem. Int. Ed.* 55(12), 3942–3946. doi:10.1002/anie.201511189

Guo, B., Cheng, X., Tang, Y., Guo, W., Deng, S., Wu, L., et al. (2022). Dehydrated UiO-66(SH)_2_: The Zr−O Cluster and its photocatalytic role mimicking the biological nitrogen fixation. *Angew. Chem. Int. Ed.* 61(13), e202117244. doi:10.1002/anie.202117244

An, K., Ren, H., Yang, D., Zhao, Z., Gao, Y., Chen, Y., et al. (2021). Nitrogenase-inspired bimetallic metal organic frameworks for visible-light-driven nitrogen fixation. *Appl. Catal. B* 292, 120167. doi:10.1016/j.apcatb.2021.120167

Gao, W., Li, X., Zhang, X., Su, S., Luo, S., Huang, R., et al. (2021). Photocatalytic nitrogen fixation of metal–organic frameworks (MOFs) excited by ultraviolet light: Insights into the nitrogen fixation mechanism of missing metal cluster or linker defects. *Nanoscale* 13(16), 7801–7809. doi:10.1039/D1NR00697E

Chen, L.-W., Hao, Y.-C., Guo, Y., Zhang, Q., Li, J., Gao, W.-Y., et al. (2021). Metal-organic framework membranes encapsulating gold nanoparticles for direct plasmonic photocatalytic nitrogen fixation. *J. Am. Chem. Soc.* 143(15), 5727–5736. doi:10.1021/jacs.0c13342

Mou, H., Wang, J., Yu, D., Zhang, D., Chen, W., Wang, Y., et al. (2019). Fabricating amorphous g-C_3_N_4_/ZrO_2_ photocatalysts by one-step pyrolysis for solar-driven ambient ammonia synthesis. *ACS Appl. Mater. Interfaces* 11(47), 44360–44365. doi:10.1021/acsami.9b16432

Feng, C., Liu, J., Li, Q., Ji, L., Wu, P., Yuan, X., et al. (2022). Fabricating Ag/PW_12_/Zr-mTiO_2_ composite via doping and interface engineering: An efficient catalyst with bifunctionality in photo- and electro-driven nitrogen reduction reactions. *Adv. Sustain. Syst.* 6(1), 2100307. doi:10.1002/adsu.202100307
